# Dynamic Thiol–ene Polymer Networks Enabled by Bifunctional Silyl Ether Exchange

**DOI:** 10.1021/acsapm.5c03887

**Published:** 2026-01-21

**Authors:** Harry E. Touloukian, Andrew D. Vargo, Clara B. Middleton, Victoria A. Pete, Matthew E. McLaughlin, Ye Yul Lee, Matthew J. Corkey, Bassil M. El-Zaatari

**Affiliations:** Department of Chemistry, 2813 Davidson College, 209 Ridge Rd, P.O. Box 5000, Davidson, North Carolina 28035, United States

**Keywords:** Covalent Adaptable Network, Dynamic Polymer, Silyl Ether Exchange, Click Chemistry, Reprocessable, Stress Relaxation

## Abstract

Dynamic polymer networks bridge the gap between traditional thermoplastics and thermosets, representing an avenue toward sustainable polymer synthesis. In this study, we utilize photoinitiated thiol–ene click chemistry to synthesize dynamic polymer networks through incorporating a series of bifunctional silyl ether alkene cross-linkers in the presence of catalytic *p*-toluene sulfonic acid. We demonstrate that the viscoelastic properties of the material, represented by its stress relaxation time constant, can be manipulated by up to 3 orders of magnitude by simple modifications in catalyst loading, amount of silyl ether cross-linker present, and/or dynamic cross-linker length. Our results show that a nonmonotonic relationship exists between stress relaxation kinetics and cross-linker length. Two representative networks were chosen to illustrate reprocessability under mild temperature conditions. These networks exhibited no loss of mechanical integrity after three reprocessing cycles. The networks can also be fully degraded in the presence of an excess of an acid catalyst.

## Graphical abstract

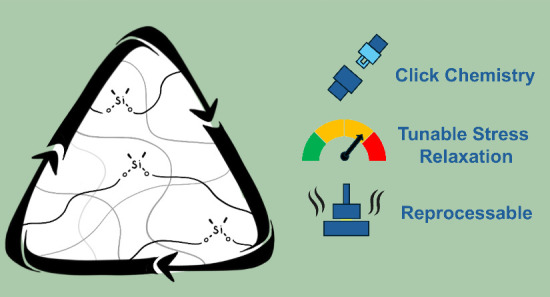

## Introduction

The demand for sustainable polymeric materials continues to grow. Within this field, cross-linked polymers that contain dynamic covalent bonds—bonds that are reversible through equilibrium-driven reactivity—are especially appealing, as they combine the reprocessability of thermoplastics with the mechanical strength of thermosets. These materials, coined Covalent Adaptable Networks (CANs),^1,2^ have garnered attention over the past couple of decades as materials that integrate permanent network stability with reversible bond exchange dynamics under specific stimuli such as heat, light, or pH.

The exchange mechanism in CANs can occur via either dissociative or associative mechanisms. Dissociative exchange mechanisms include reactions such as reversible Diels–Alder additions,^3–6^ thioacetal exchanges,^7^ dynamic imine exchanges,^8,9^ and hindered urea bond exchanges.^10,11^ Associative exchange mechanisms, which maintain constant cross-link density, define vitrimers; these reactions encompass transesterification reactions,^12–14^ olefin metathesis,^15^ dioxaborolane exchange,^16,17^ vinylogous urethane exchange,^18–21^ and dithioalkylidene exchanges.^22–24^ These molecular exchange reactions provide the fundamental basis for the unique properties of dynamic networks, enabling materials that can undergo stress relaxation, self-healing, reshaping, and reprocessing while maintaining network integrity through the precise control of bond formation and breaking kinetics.

Silicone polymers that contain Si–O bonds throughout their backbone are typically known for their desirable macromolecular properties, such as enhanced chemical and thermal stability.^25–27^ Within the past decade, there have been efforts to synthesize Si–O-based CANs. Many of these dynamic polymers are based on cross-linked polydimethylsiloxane (PDMS) networks incorporating dynamic covalent cross-links that undergo various dynamic exchanges.^12,28–33^ Recently, increasing attention has been paid to CANs that undergo intrinsic siloxane or silyl ether exchanges. Toward this goal, Guan and co-workers synthesized polymer networks that exhibited silyl ether metatheses enabled by hydroxyl groups and Bronsted-acid catalyzed exchanges.^34,35^ Rapid siloxane exchanges that were hydroxy and fluoride mediated were furthermore developed by Du Prez and Guan, respectively.^36,37^ Johnson and co-workers developed dynamic polydicyclopentadiene thermoset networks enabled by dynamic bifunctional silyl ether which was catalyzed by octanoic acid,^38^ while Pierce et al. utilized dynamic silyl ether exchanges to invoke degradability in melamine-based adhesives.^39^ Furthermore, silyl ether bonds have been utilized for the synthesis of reprocessable epoxy-based composites,^40^ and as a dynamic motif for PDMS vitrimers.^41^

Here, we take advantage of thiol–ene click chemistry to synthesize CANs via bifunctional silyl ether exchanges catalyzed by *p*-toluenesulfonic acid (pTSA). Importantly, this synthetic platform is advantageous due to its operational simplicity. That is, the dynamic bonds are introduced via orthogonal and photoinduced click reactions. As a result, these dynamic networks can be prepared in bulk and at room temperature. We show that the rate of stress relaxation of these networks can be tuned and modulated based on the length of the bifunctional silyl ether monomer used, the amount of catalyst in the network, and the ratio of dynamic to static cross-linkers in the network. We demonstrate that our materials can be efficiently reprocessed under mild temperature conditions (∼70 °C) without exhibiting any loss in thermomechanical or viscoelastic performance after three cycles. Finally, the networks exhibit acid-triggered degradability in under 24 h.

## Materials and Methods

### Bifunctional Silyl Ether Cross-Linker Synthesis

All reactions were performed in dichloromethane (DCM). Chemicals were obtained from MilliporeSigma, TCI Chemicals, Oakwood Chemicals, and Fisher Scientific. The four cross-linkers were synthesized via substitution reactions of dimethyldichlorosilane with alkenols (Figure 1b). This general synthetic strategy was adapted from methodology reported by Husted et al., with slight modifications.^38^ Synthetic details are provided below.

**Figure 1:**
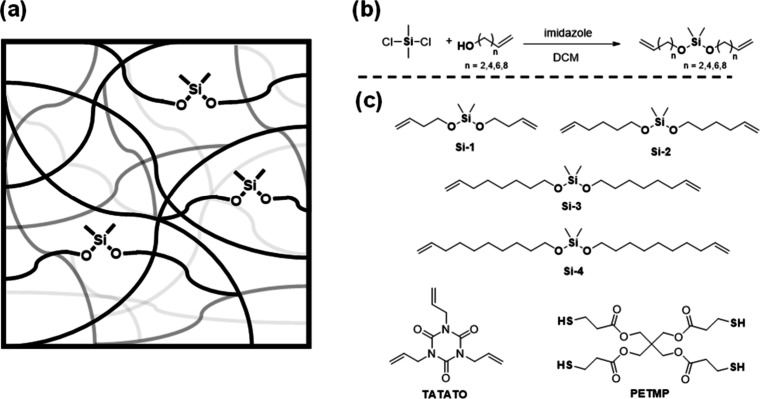
(a) Schematic representation of the cross-linked polymer network that contains bifunctional silyl ether moieties. (b) General synthetic procedure for the cross-linkers used in the study. (c) Chemical structures of different cross-linkers and monomers used in the study.

### **Si-1** Synthesis

First, a 1.36 g (20 mmol, 2 equiv) portion of imidazole and 1.74 mL (1.44 g, 20 mmol, 2 equiv) of 3-buten-1-ol were dissolved in 20 mL of dichloromethane. The reaction mixture was cooled to 0 °C in an ice bath. Next, 1.22 mL (1.29 g, 10 mmol) of dichlorodimethylsilane was added dropwise over 5 min to the reaction mixture. After the formation of a white precipitate (∼20 min), the reaction was removed from the ice and allowed to react at room temperature and stir for another 4 h. The reaction mixture was vacuum filtered to remove the imidazole salt precipitate, and the remaining dichloromethane was removed via rotary evaporation. The resulting mixture was then diluted with 100 mL of hexanes and washed with 3 × 100 mL of brine. The solution was dried with sodium sulfate in a vacuum filter and was subsequently concentrated via rotary evaporation until the concentrated product was isolated, yielding 1.24 g (6.20 mmol, 62.0%) of the product as a clear oil.

^1^H NMR (400 MHz, CDCl_3_) δ 5.82 (ddt, *J* = 17.1, 10.2, 6.8 Hz, 2H), 5.15–4.99 (m, 4H), 3.72 (t, *J* = 6.9 Hz, 4H), 2.31 (qt, *J* = 7.0, 1.4 Hz, 4H), 0.13 (s, 6H). ^13^C NMR (101 MHz, CDCl_3_) δ 134.94, 116.38, 61.83, 36.91, −3.35. HMRS (APCI): expected, 201.1305; found, 201.1305.

### **Si-2** Synthesis

First, a 1.36 g (20 mmol, 2 equiv) portion of imidazole and 2.00 g (20 mmol, 2 equiv) of 5-hexen-1-ol were dissolved in 20 mL of dichloromethane. The reaction was cooled to 0 °C in an ice bath. Next, 1.22 mL (1.29 g, 10 mmol) of dichlorodimethylsilane was added dropwise over 5 min to the reaction mixture. After the formation of a white precipitate (∼20 min), the reaction was removed from the ice and allowed to react at room temperature and stir for another 4 h. The reaction mixture was vacuum filtered to remove the imidazole salt precipitate, and the remaining dichloromethane was removed via rotary evaporation. The resulting mixture was then diluted with 100 mL of hexanes and washed with 3 × 100 mL brine. The solution was dried with sodium sulfate in a vacuum filter and subsequently concentrated via rotary evaporation until the concentrated product was isolated, yielding 2.07 g (8.05 mmol, 80.5%) of the product as a clear oil.

^1^H NMR (400 MHz, CDCl_3_) δ 5.79 (ddt, *J* = 16.9, 10.1, 6.6 Hz, 2H), 5.05–4.88 (m, 4 H), 3.67 (t, *J* = 6.6 Hz, 4H), 2.07 (qt, *J* = 7.3, 7.1 Hz, 4H), 1.57 (dq, *J* = 8.5, 6.5 Hz, 4H), 1.51–1.36 (m, 4H), 0.11 (s, 6H). ^13^C NMR (101 MHz, CDCl_3_) δ 139.96, 114.61, 62.49, 33.66, 32.18, 25.28, −3.06. HMRS (APCI): expected, 257.1931; found, 257.1928

### **Si-3** Synthesis

First, a 1.36 g (20 mmol, 2 equiv) portion of imidazole and 2.56 g (20 mmol, 2 equiv) of 7-octen-1-ol were dissolved in 20 mL of dichloromethane. The reaction was cooled to 0 °C in an ice bath. Next, 1.22 mL (1.29 g, 10 mmol) of dichlorodimethylsilane was added dropwise to the reaction mixture over 5 min. After the formation of a white precipitate (∼20 min), the reaction was removed from the ice and allowed to react at room temperature and stir for another 4 h. The reaction mixture was vacuum filtered to remove the imidazole salt precipitate, and the remaining dichloromethane was removed via rotary evaporation. The resulting mixture was then diluted with 100 mL of hexanes and washed with 3 × 100 mL brine. The solution was dried with sodium sulfate in a vacuum filter and subsequently concentrated via rotary evaporation until the concentrated product was isolated, yielding 2.75 g (8.81 mmol, 88.1%) of the product (**Si-3**) as a faint yellow oil.

^1^H NMR (400 MHz, CDCl_3_) δ 5.81 (ddt, *J* = 16.9, 10.1, 6.6 Hz, 2H), 5.00–4.91 (m, 4H), 3.66 (t, *J* = 6.7 Hz, 4H), 2.04 (qt, *J* = 7.2, 7.0 Hz, 4H), 1.61–1.49 (m, 4H), 1.45–1.28 (m, 12H), 0.11 (s, 6H). ^13^C NMR (101 MHz, CDCl_3_) δ 138.94, 114.02, 62.36, 33.57, 32.38, 28.74, 28.73, 25.51, −3.36. HRMS (APCI): expected, 313.2557; found, 313.2565

### **Si-4** Synthesis

First, 1.36 g (20 mmol, 2 equiv) of imidazole and 3.13 g (20 mmol, 2 equiv) of 9-decen-1-ol were dissolved in 20 mL of dichloromethane. The reaction was cooled to 0 °C in an ice bath. Next, 1.22 mL (1.29 g, 10 mmol) of dichlorodimethylsilane was added dropwise to the reaction mixture over 5 min. After the formation of a white precipitate (∼20 min), the reaction was removed from the ice and allowed to react at room temperature and stir for another 4 h. The reaction mixture was vacuum filtered to remove the imidazole salt precipitate, and the remaining dichloromethane was removed via rotary evaporation. The resulting mixture was then diluted with 100 mL of hexanes and washed with 3 × 100 mL brine. The solution was dried with sodium sulfate in a vacuum filter and subsequently concentrated via rotary evaporation until the concentrated product was isolated, yielding 2.73 g (7.40 mmol, 74.0%) of the product (**Si-4**) as a clear oil.

^1^H NMR (400 MHz, CDCl_3_) δ 5.81 (ddt, *J* = 16.9, 10.2, 6.7 Hz, 2H), 5.07–4.89 (m, 4H), 3.66 (t, *J* = 6.8 Hz, 4H), 2.12–1.98 (m, 4H), 1.55 (q, *J* = 7.0 Hz, 4H), 1.43–1.26 (m, 20H), 0.12 (s, 6H). ^13^C NMR (101 MHz, CDCl_3_) δ 139.36, 114.27, 62.73, 33.95, 32.77, 29.63, 29.55, 29.24, 29.08, 25.96, −3.03. HMRS (APCI): expected, 369.3183; found, 369.3188

### Network Formation

All polymer syntheses followed a similar procedure but varied based on type and quantity of cross-linker in the system as well as amount of para-toluenesulfonic acid (pTSA). Briefly, pentaerythritol tetrakis­(3-mercaptopropionate) (**PETMP**) was combined with silyl ether cross-linker (**Si-1**, **Si-2**, **Si-3**, or **Si-4**) and 1,3,5-triallyl-1,3,5-triazine-2,4,6­(1H,3H,5H)-trione (**TATATO**) and the appropriate wt % of pTSA, calculated as a function of total mass of monomers added. The photoinitiator 2,2-dimethoxy-1,2-diphenylethan-1-one was always added at 3 wt %. The solution was mixed thoroughly using a Hauschild 150.1 FVZ-K speedmixer (3500 rpm for 5 min). It was then placed into a 10 mm diameter × 1.8 mm deep silicone well (Ladd Research Industries). Once in the mold, the samples were cured under 365 nm light using a WheeLED lamp (Mightex) until the mixture polymerized as a homogeneous, cross-linked thermoset (approximately 30 min). The samples were then postcured at 90 °C in an oven for 90 min to ensure full reactivity.

### Stress Relaxation Experiments

The stress relaxation behavior of the networks was studied by using a strain-controlled Anton Paar rheometer (MCR302e). Stress relaxation measurements were performed at the described temperatures at 1% strain. Under a parallel plate geometry (with a diameter of 8 mm), the samples were subjected to a normal force between 3 and 6 N before the start of each test. It is important to note that all measurements took place in the linear viscoelastic regime, as determined by amplitude sweeps.

### Reprocessing

Polymer samples were cut into tiny pieces and placed between two sheets of polyamide film using 0.3 mm-thick spacers. The chopped samples were then placed under 7 MPa at 70 °C using an MSE PRO 24-Ton Manual Hot Press for 45 min to obtain the reprocessed polymer films.

### Stress–Strain Studies

Tensile tests were performed on rectangular samples with approximate dimensions of ∼10 × 5 × 0.3 mm on both virgin and reprocessed samples using a TA Instruments DMA Q850. All tests were conducted at 30 °C with a preload force of 0.5 mN and a strain rate of 2.0% per minute. Young’s modulus was calculated from the slope of the linear portion of the stress–strain curve between 0.5% and 10% strain. The strain at break was determined at the strain value corresponding to sample breakage at its maximum. The tensile strength was the maximum stress reached prior to sample breakage.

### Polymer Degradation

The synthesized polymer networks were sliced into 20 mg samples with similar dimensions and were fully immersed in a solution of 1 M pTSA in tetrahydrofuran (THF). The networks were monitored over the course of 24 h, or until the solid sample had completely degraded into the solution. The initial and final degradation solutions were furthermore evaluated using ^1^H NMR spectroscopy.

## Results and Discussion

The use of click chemistry for the synthesis of polymer networks is advantageous due to its selectivity, orthogonality and ability to proceed under mild conditions.^42,43^ Thiol–ene ‘click’ chemistry involves the reaction between thiol and alkene groups, yielding alkyl sulfides. Photo-cross-linking through thiol–ene reactions is especially attractive owing to its rapid kinetics and relatively high oxygen tolerance.^44^ Due to these advantages, this reaction has been employed in the synthesis of dynamic polymers and CANs by cross-linking monomers that incorporate dynamic bonds.^45–53^ We aimed to utilize thiol–ene photopolymerizations to synthesize polymer networks that contained dynamic bifunctional silyl ether groups (Figure 1a). To this end, four different bifunctional alkenes with a silyl ether core were synthesized through a simple nucleophilic substitution reaction between dimethyldichlorosilane and a primary alkenyl alcohol, as shown in Figure 1b. These cross-linkers are depicted in Figure 1c.

The silyl ether cross-linkers were then mixed with pentaerythritol tetrakis­(3-mercaptopropionate) (**PETMP**) and 1,3,5-triallyl-1,3,5-triazine-2,4,6­(1H,3H,5H)-trione (**TATATO**) (Figure 1c), so that there was an equimolar amount of thiols and alkenes present. The photopolymerization process occurred in the presence of 3 wt % of the photoinitiator 2,2-dimethoxy-2-phenylacetophenone under 365 nm light. Networks are named by the silyl ether cross-linker used, followed by the mol % of alkenes coming from the silyl ether cross-linker compared to thiol groups (between 0 and 100) and the weight percent of pTSA added into the network in parentheses. For example, a network that is formed with **PETMP** and 25 mol % **Si-2**, 75 mol % **TATATO** and 1 wt % pTSA will have the following convention: **Si-2­(25–1)**.

To test the dynamic nature of the polymer network, we examined how the incorporation of silyl ether cross-linkers into this network influenced its thermorheological properties. Hence, stress relaxation experiments were performed using shear rheology at 100 °C on two polymer networks; the first network contained no silyl ether cross-linker and was formulated with only **TATATO** and **PETMP**, which served as the control, whereas the second network contained 50 mol % of **Si-2** with **TATATO**. Then 0.5 wt % of pTSA was added to both networks before curing. The control sample showed no significant relaxation after 70 min while the **Si-2­(50–0.5)** network relaxed over 60% of its initial stress during the same time interval, as shown in Figure 2a. This result confirms that the observed network responsiveness arises specifically from the incorporation of the dynamic silyl ether cross-linker in the presence of a sulfonic acid catalyst. Next, the amount of acid catalyst was varied to assess the sensitivity of the exchange to acid concentration. Specifically, we sought to compare the characteristic relaxation time, τ, between the samples. Here, τ was calculated using a stretched exponential model shown in eq 1:

1 $$\frac{G\left(t\right)}{G_{0}}=e^{{\left(-\frac{t}{\tau }\right)}^{\beta }}$$

where *G*(*t*) represents the relaxation modulus at any given time, *G*_0_ is the initial relaxation modulus, *t* is time, and β is the stretching exponent parameter that depicts the broadness of the relaxation process.

**Figure 2:**
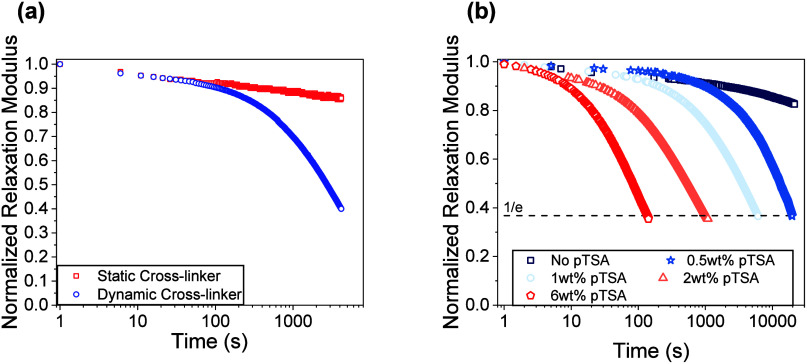
(a) Comparison of the normalized stress relaxation modulus at 100 °C between two polymer networks: the static network contained no **Si-2** and the dynamic network contained 50 mol % of **Si-2** and 0.5 wt % pTSA. (b) Representative normalized stress relaxation profiles at 80 °C depicting the influence of different amounts of pTSA catalyst between 0 and 6 wt %.

The appearance of a single dominant relaxation mode, as shown by the continuous relaxation spectra (see the Supporting Information), suggests that the main avenue of stress relaxation comes from a dominant relaxation event: the bifunctional silyl ether bond exchange. Regardless, we utilized a stretched-exponential analysis as the β values mostly ranged between 0.6 and 0.8. These values indicate a broadening that cannot be captured by a single-exponential Maxwell model.^54,55^

Five identical polymer network samples were synthesized that varied in weight percent of pTSA added prior to polymerization: **Si-2­(50–0)**, **Si-2­(50–0.5)**, **Si-2­(50–1)**, **Si-2­(50–2)**, and **Si-2­(50–6)**. The stress relaxation behavior of the samples at 80 °C displayed a strong dependence on the amount of catalyst present in the network. **Si-2­(50–0)**, which contained no added catalyst, showed no significant stress relaxation after over 5 h. Alternatively, **Si-2­(50–0.5)**, **Si-2­(50–1)**, **Si-2­(50–2)**, and **Si-2­(50–6)** all exhibited a monotonic decrease in τ with over 2 orders of magnitude difference between **Si-2­(50–0.5)** and **Si-2­(50–6)** at the same temperature (Figure 2b). The addition of pTSA up to 6 wt % did not significantly influence the rubbery plateau modulus nor the *T*_g_ of the samples (see Figure S51 and Table S19), implying that the relaxation behavior can be directly attributed to the concentration of catalyst in the system.

Another important parameter in evaluating the thermorheological behavior of dynamic networks is the flow activation energy (*E*_a,flow_). This measurement quantifies the temperature dependence of the material’s viscoelastic relaxation and serves as a key parameter for comparing the polymer’s viscoelastic sensitivity to temperature changes. The flow activation energies can reflect the energy barrier associated with the dynamic bond exchange reactions. τ is related to *E*_a,flow_ of the polymer through an Arrhenius relationship shown in eq 2:

$$\tau =Ae^{E_{a,flow}/RT}$$ 2

where *R* is the ideal gas constant, *T* is the temperature (in Kelvin), and *A* is a pre-exponential factor. *E*_a,flow_ values for the three networks **Si-2­(50–1)**, **Si-2**(**50–2)**, and **Si-2­(50–6)** were calculated by performing stress relaxation experiments on the samples in a temperature window between 60 and 100 °C as shown in Figure 3a. A very slight decrease in *E*_a,flow_ was observed between the samples where **Si-2­(50–1)** had an average *E*_a,flow_ of 97 kJ/mol, whereas **Si-2­(50–6)**’s *E*_a,flow_ decreased to 89 kJ/mol (Figure 3b, Table 1 entry 1). While an increase in catalyst loading has been shown to decrease the flow activation energy of dynamic systems which can be attributed to increased catalytic efficiency,^56–58^ the observed averages do not differ significantly within the limits of statistical uncertainty, as determined by an unpaired *t*-test. Hence, the influence of catalyst loading on the flow activation energy in these networks is limited in magnitude.

**Figure 3:**
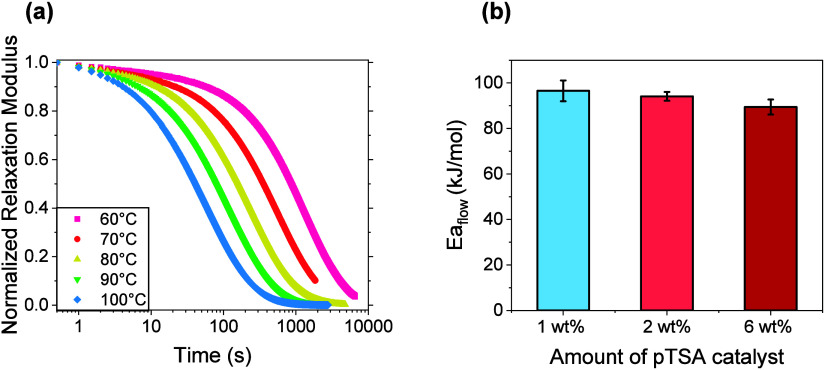
(a) Representative normalized stress relaxation profiles of **Si-2­(50–6)** between 60 and 100 °C. (b) Calculated flow activation energies of **Si-2­(50–1)**, **Si-2­(50–2)**, and **Si-2­(50–6)**. Error bars represent standard error from triplicates.

**Table 1:** Viscoelastic Properties (τ at 100 °C and Flow Activation Energies) for Synthesized Polymer Networks^a^

entry	network	τ_100 °C_ (s)	*E*_a,flow_ (kJ/mol)
1	**Si-2(50–6)**	44 ± 13	89 ± 3
2	**Si-2(10–6)**	1200 ± 98	82 ± 8
3	**Si-2(25–6)**	110 ± 29	88 ± 1
4	**Si-2(60–6)**	26 ± 2	80 ± 1
5	**Si-1(50–6)**	32,000 ± 11,000	115 ± 5
6	**Si-3(50–6)**	76 ± 10	91 ± 3
7	**Si-4(50–6)**	200 ± 48	93 ± 2

a Uncertainty values represent standard error from triplicates.

The amount of static to dynamic cross-linker ratio was moreover manipulated to probe its influence on the viscoelastic behavior of the polymer networks; **Si-2­(10–6)**, **Si-2­(25–6)**, and **Si-2­(60–6)** were synthesized such that the amount of catalyst remained the same. **Si-2** loadings above 60 mol % did not yield complete polymer networks in the presence of 6 wt % pTSA. This is most likely attributed to enhanced exchange kinetics, where the catalyst accelerates both bond formation and exchange, hence suppressing higher molecular weight formation during the photopolymerization process. We also observed that the addition of **TATATO** to the polymer mixture improved the solubility of the bulk solution. The extent of stress relaxation was directly influenced by the relative amount of permanent versus dynamic cross-links, where **Si-2­(10–6)** showed a 10-fold and over 40-fold increase in τ_100 °C_ when compared to **Si-2­(25–6)** and **Si-2­(60–6)**, respectively, as shown in Table 1 (entries 2, 3, and 4). While the decrease in silyl ether content leads to slower relaxation kinetics due to a decrease in dynamic functional groups throughout the network, it should also be noted that the *G*_p_ increased with decreasing silyl ether content, which could be further contributing to the retardation in relaxation kinetics (Figure S51).

Flow behavior can be strongly influenced by factors such as network stiffness and cross-linking density. In order to evaluate these factors on network viscoelastic properties, a series of four bifunctional silyl ether alkene cross-linkers was synthesized, each differing by an additional two methylene units on either side (Figure 1c). Four different polymer networks were synthesized with 50 mol % of the silyl ether cross-linker and 6 wt % pTSA: **Si-1­(50–6)**, **Si-2­(50–6)**, **Si-3­(50–6)**, and **Si-4­(50–6)**. We hypothesized that as cross-linker length increased from *n* = 2 to *n* = 8 (Figure 1b), stress relaxation would be faster due to the decrease in network constraint from lowered cross-linking density, facilitating the silyl ether exchange arising from increased mobility and flexibility in the network. Interestingly, the relaxation dynamics were fastest in **Si-2­(50–6)**, followed closely by **Si-3­(50–6)** and then **Si-4­(50–6)**, and slowest in **Si-1­(50–6)** (Table 1 entries 1, 5, 6, and 7). The stress relaxation terminus of the exchange reaction may vary depending on cross-link density, as depicted in chain length, whereby both expected monotonic^59^ and nonmonotonic^60^ trends have been observed. The extremely slow relaxation kinetics from **Si-1­(50–6)** (τ_100 °C_ = 32,000 ± 11,000 s) can be in part attributed to its higher cross-linking density and conversely reduced catalyst diffusivity. Similar trends were observed in flow activation energies where **Si-1­(50–6)** had a significantly higher *E*_a,flow_ at 115 ± 5 kJ/mol whereas the others ranged between 89 and 93 kJ/mol (Table 1).

In order to assess whether the slow relaxation in **Si-1­(50–6)** indeed arises from bulk network effects rather than inherent molecular kinetic differences, we then performed comparative kinetic studies on model compounds with the three cross-linkers **Si-1**, **Si-2**, and **Si-4**. These molecules were reacted with dimethyldimethoxysilane as shown in Figure 4a in the presence of 1.5 mol % pTSA in benzene-d_6_. The reaction progress was monitored by ^1^H NMR spectroscopy. Specifically, the decrease in the intensity of hydrogens corresponding to the dimethyl silyl peaks and appearance of a new intermediate peak representing the dimethyl silyl peaks for the mixed product (Figures 4b and 4c). For all three cross-linkers, the mixed product was formed within 5 min of mixing at room temperature, indicating catalytic efficiency and highlighting the speed of the exchange reaction (Figure 4d and Figures S53–S55). Equilibrium was furthermore reached within ∼30 min of reaction time for all samples. In the absence of pTSA, no product was observed after 24 h, showcasing the dependence of the reaction on the acid catalyst. Importantly, however, all three cross-linkers exhibited similar reactivity on a small molecule scale. This result further supports the claim that the difference in stress-relaxation times in the networks is dependent on macromolecular structure.

**Figure 4:**
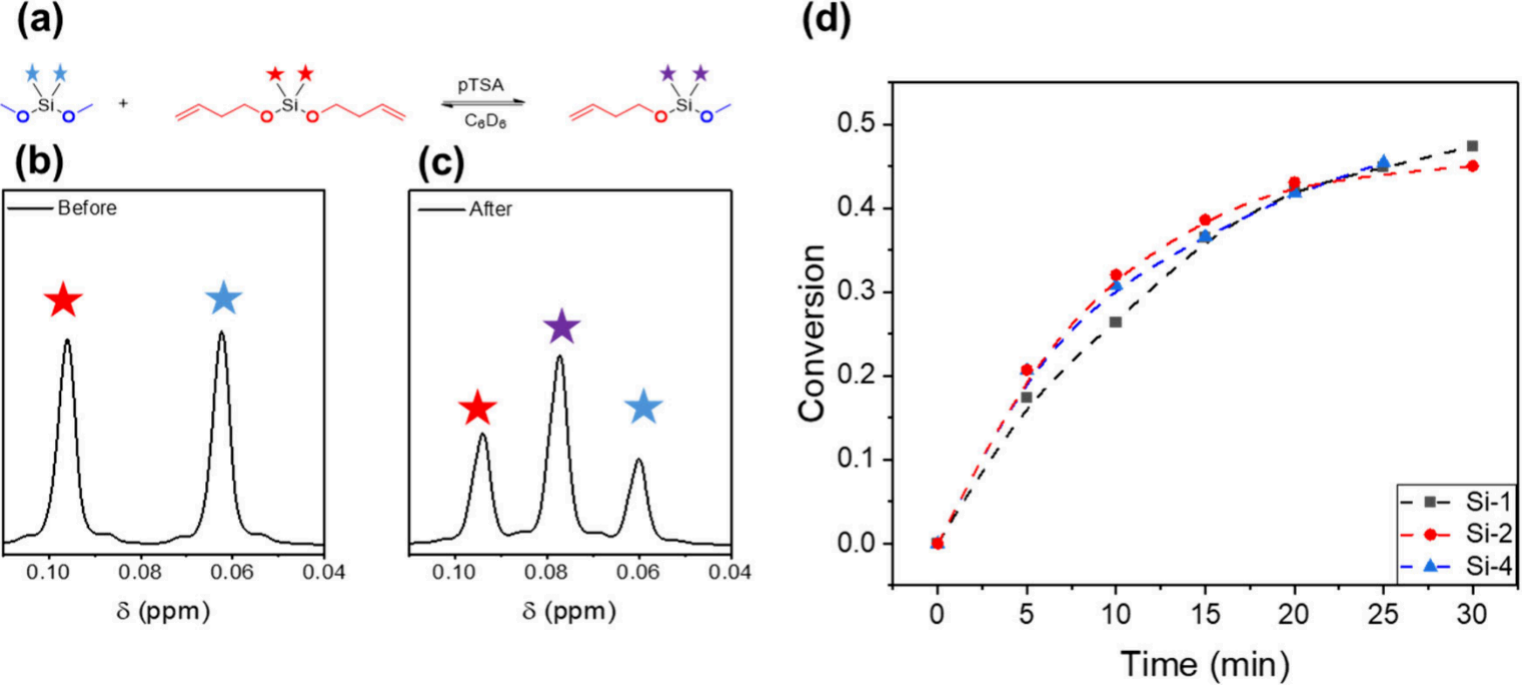
(a) Reaction scheme between dimethoxydimethylsilane and **Si-1** utilized for small molecule kinetics. (b) ^1^H NMR spectrum between 0.04 and 0.12 ppm for the reaction before the addition of pTSA. (c) ^1^H NMR spectrum between 0.04 and 0.12 ppm after pTSA was added showcasing the appearance of an intermediate product peak. (d) Conversion kinetics for **Si-1**, **Si-2**, and **Si-4** reacting with diemthoxydimethylsilane with 1.5 mol % pTSA.

To further probe the role of the slowing of stress relaxation kinetics between **Si-1­(50–6)** and **Si-2­(50–6)**, diallyl carbonate (DAC) was utilized as cocrosslinker with either **Si-1** or **Si-2** instead of **TATATO**. The replacement of **TATATO** with DAC at the same mole percentage of alkene functionality significantly decreases the modulus and hence cross-linking density of both networks. Stress relaxation experiments were conducted on each synthesized network, and the results show a smaller τ value at 100 °C when compared to using **TATATO** as a co-cross-linker, indicating that lowering the cross-linking density can speed up the exchange process in the bulk polymer system. Interestingly, the **Si-2**/DAC network still exhibited a significantly lower τ value (<10 s) when compared to **Si-1**/DAC which reached τ at around 1500 s (Figure S52). Taken together, these results further support the claim that the retardation in relaxation kinetics in **Si-1** networks is, in part, dependent on cross-linking density. However, the vast difference (2 to 3 orders of magnitude) in calculated τ values between **Si-1** and **Si-2** networks might be due to other reasons as well. For example, shorter spacers may impair the accessibility of the catalytic acid, which would reduce the effective interchain exchange events in the bulk polymer. Other factors such as network inhomogeneity, saturation of effective catalyst loading, and constraints on the exchange geometry in the network could also play a role. Importantly, however, these results highlight how subtle changes in polymer design in CANs can lead to large and sometimes unexpected viscoelastic differences that cannot be captured from small molecule reactivity.

When comparing the τ_100 °C_ values of **Si-2­(50–6)** to **Si-3­(50–6)** and **Si-4­(50–6)**, the mole fraction of the reactive alkene groups of the bifunctional silyl ether is the same at 50 mol %. However, increasing the spacer length effectively lowers the local density of the O–Si–O groups within the network, which may contribute to the slower relaxation observed for longer cross-linkers. This can explain why **Si-2­(50–6)** exhibits the fastest relaxation kinetics even though it has a higher cross-linking density than **Si-3­(50–6)** and **Si-4­(50–6)**. This dichotomy depicts a nonmonotonic behavior where intermediate cross-linker lengths yield the fastest relaxation behavior. It should be noted that additional factors, such as catalyst solubility, increasing hydrophobicity of the networks with increasing chain length, and network homogeneity, could also be playing a role.

The reprocessability of the dynamic thiol–ene networks was studied through compression molding using a hot press. Specifically, both **Si-2­(50–6)** and **Si-3­(50–6)** were cut and torn into several pieces and then placed in a hot press at 70 °C under 7 MPa of force for 45 min, after which a uniform mass was obtained as shown in Figure 5a. Here, we chose a low reprocessing temperature to highlight the polymer’s ability to undergo dynamic exchange well below the temperatures typically reported for similar chemistries.^34–38,40,41^ The networks’ thermomechanical properties were measured prior to reprocessing. The polymers were then subjected to three reprocessing cycles before the thermomechanical properties were remeasured. In all cases the virgin and reprocessed networks exhibit statistically similar Young’s moduli, tensile strength, and strain at break before and after 3x reprocessing, as shown in Figures 5b–5d, determined from stress–strain experiments. Furthermore, the glass transition temperatures of **Si-2­(50–6)** and **Si-3­(50–6)** were measured before and after reprocessing. The glass transition remained relatively unchanged for both samples (Table S25). Taken together, these results indicate that the material retains its mechanical properties after reprocessing under mild conditions. The viscoelastic properties of the reprocessed sample, **Si-2­(50–6)**, were then tested through a stress relaxation experiment at 100 °C. The reprocessed sample shows similar relaxation behavior when compared to the original sample (τ = 46 s).

**Figure 5:**
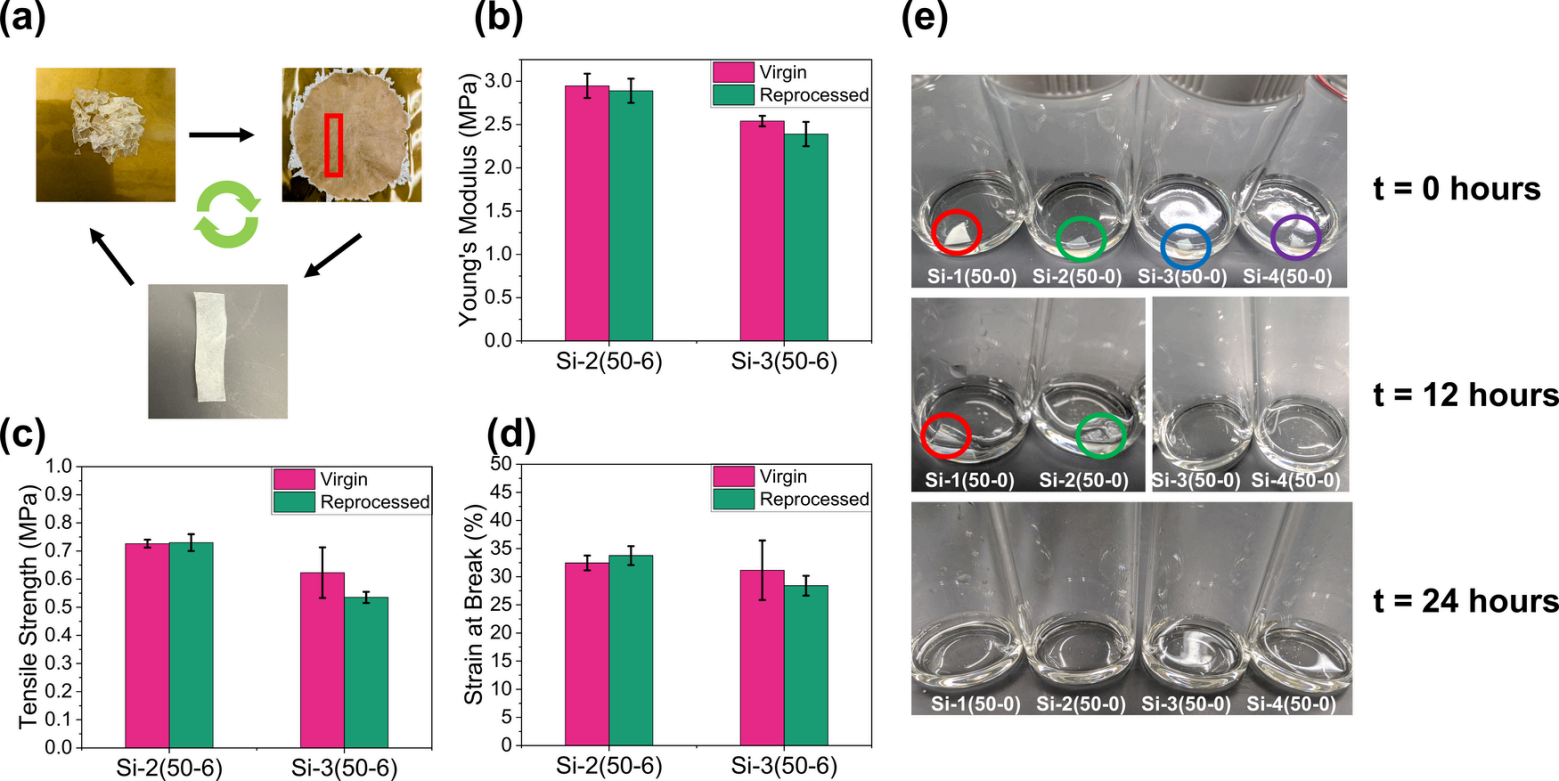
(a) The reprocessing cycle of the CAN. Following arrows from left to right: Chopped polymer sample before reprocessing, polymer sample postreprocessing, rectangular sample cut from reprocessed sample. Comparison in (b) Young’s modulus, (c) tensile strength, and (d) strain at break from stress–strain tests between virgin samples and 3x reprocessed samples **Si-2­(50–6)** and **Si-3­(50–6)**. (e) Acid-catalyzed hydrolysis of silyl-ether-containing networks when immersed in a 1 M pTSA THF solution. Circles are used to guide the eye to polymer network samples in solution.

The hydrolysis of polymers containing silyl ester bonds in acidic solutions has been well reported recently.^61–65^ We sought to test the ability of our networks to degrade in acidic media. Four silyl-ether containing networks with no added catalyst, **Si-1­(50–0)**, **Si-2­(50–0)**, **Si-3­(50–0)**, and **Si-4­(50–0)**, were cut into a small piece with similar geometries and immersed in a 1 M pTSA solution in THF. As shown in Figure 5c, all networks can be degraded within 24 h at room temperature under these conditions. Interestingly, **Si-3­(50–0)** and **Si-4­(50–0)** degraded the fastest within 12 h, whereas **Si-2­(50–0)** and **Si-1­(50–0)** fully degraded after 24 h (Figure 5c). Hence, the degradation kinetics would appear to be dependent on cross-linking density. In other words, the networks that have a lower cross-linking density (**Si-3­(50–0)** and **Si-4­(50–0)**) are able to swell to a greater extent and undergo catalytic hydrolysis faster. The networks with the higher cross-linking density (**Si-1­(50–0)** and **Si-2­(50–0)**) had more limited swelling, restricting both solvent uptake and transport through the matrix. ^1^H NMR analysis confirms the appearance of hydrolyzed silyl ethers (Figure S61). Polymers with only static cross-linker did not degrade after 72 h (see the Supporting Information). These findings illustrate the delicate balance of acid catalysis in our dynamic networks, where the incorporation of modest amounts of pTSA enables reversible exchanges without compromising mechanical integrity, whereas immersion in excess acid triggers full degradation.

## Conclusion

In this work, dynamic thiol–ene CANs were synthesized by incorporating bifunctional silyl ether alkene cross-linkers in the presence of *p*-toluene sulfonic acid. The rate of stress relaxation can be accelerated by modest increases in catalyst amount and the mol % of the silyl ether cross-linker in the network formulation without influencing the flow activation energy to a large extent. The cross-linker length further influences the viscoelastic properties of the network. The shortest cross-linker **Si-1** exhibited extremely slow relaxation kinetics when incorporated into the polymer network, even at high catalytic loadings, due in part to an increase in cross-linking density and subsequent catalytic immobility. **Si-2** showed the fastest stress relaxation, followed by **Si-3** and **Si-4**, respectively. The capability of these networks to reform and reshape via compression molding was demonstrated through the reprocessability of **Si-2­(50–6)** and **Si-3­(50–6)** at 70 °C. The networks maintained their thermomechanical and viscoelastic properties after three reprocessing cycles. Finally, when the sulfonic acid catalyst is utilized in a large excess, it drives complete network degradation within 24 h for all samples.

## Supplementary Material



## Notes

Stress relaxation data, activation energy plots, H and C NMR spectra of synthesized compounds, polymer synthesis, small molecule kinetics data, frequency sweep, DSC data, FTIR conversion, stress-strain data for reprocessed samples and degradation NMR data (PDF)
