# Supplementary material for: Dynamic Thiol–ene Polymer Networks Enabled by Bifunctional Silyl Ether Exchange
Source: ACS Appl Polym Mater. 2026 Jan 21;8(3):1795–803. doi: 10.1021/acsapm.5c03887 (PMC12910552; doi:10.1021/acsapm.5c03887)
Supplement: Supplementary file 1 [file ap5c03887_si_001.pdf]

Supporting Information

for

Dynamic Thiol-ene Polymer Networks Enabled by Bifunctional Silyl Ether Exchange

Harry E. Touloukian‡, Andrew D. Vargo‡, Clara B. Middleton, Victoria A. Pete, Matthew E. McLaughlin, Ye Yul Lee, Matthew J. Corkey, Bassil M. El-Zaatari*

Department of Chemistry, Davidson College, Davidson, NC 28035, USA

*email: baelzaatari@davidson.edu

Contents

Stress Relaxation Data S2

Si-1(50-6) S2

Si-2(50-1) S4

Si-2(50-2) S6

Si-2(50-6) S8

Si-2(10-6) S12

Si-2(60-6) S14

Si-3(50-6) S16

Si-4(50-6) S18

Continuous Relaxation Spectra S20

Activation Energy Plots S21

Varying Cross-linker Length S21

Varying wt% pTSA S22

Varying dynamic: static cross-linker ratio S22

NMR Spectra S23

Polymer Synthesis S31

Polymer Conversion S32

Frequency Sweep Data S37

Diallyl Carbonate Stress Relaxation Data S38

Small molecule kinetics S39

Reprocessed Samples Data S43

Degradation Data S47

Stress Relaxation Data

Si-1(50-6)

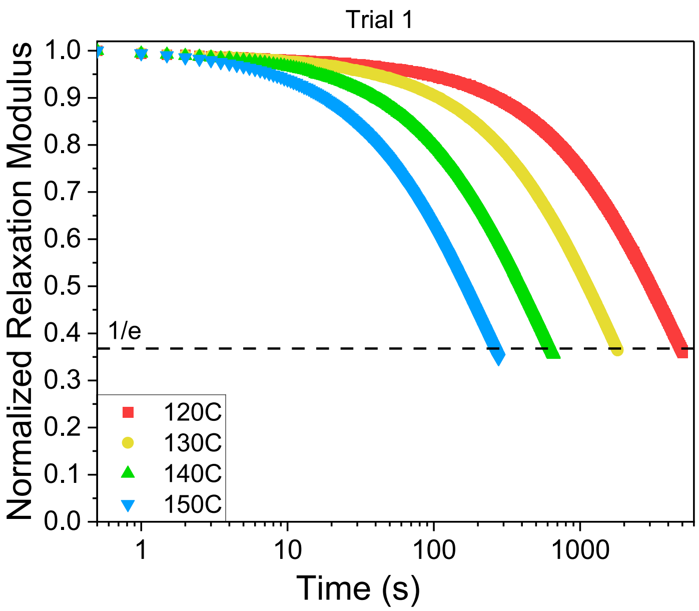

Figure S1. Normalized stress relaxation as a function of time at temperatures ranging from 120 to 150 ºC for **Si-1(50-6)** sample 1.

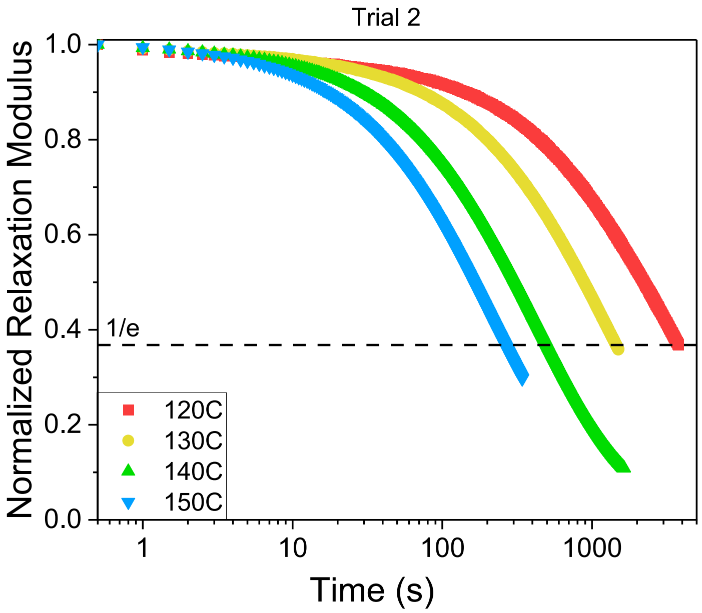

Figure S2. Normalized stress relaxation as a function of time at temperatures ranging from 120 to 150 ºC for **Si-1(50-6)** sample 2.

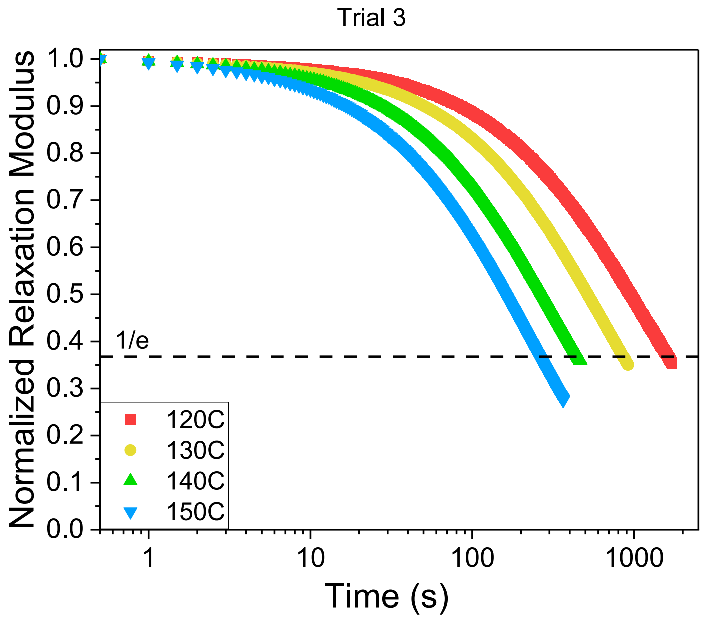

Figure S3. Normalized stress relaxation as a function of time at temperatures ranging from 120 to 150 ºC for **Si-1(50-6)** sample 3.

Table S1. τ values calculated for Si-1(50-6) at various temperatures

Temperature (°C)	$\tau_{trial\ 1}$ (s)	$\tau_{trial\ 2}$ (s)	$\tau_{trial\ 3}$ (s)
120	4873	3777	1587
130	1781	1445	842
140	628	518	440
150	255	261	256

Table S2. β parameters calculated for Si-1(50-6) at various temperatures

Temperature (°C)	$\beta_{trial\ 1}$	$\beta_{trial\ 2}$	$\beta_{trial\ 3}$
120	0.78	0.70	0.73
130	0.81	0.75	0.77
140	0.81	0.73	0.78
150	0.82	0.79	0.79

Si-2(50-1)

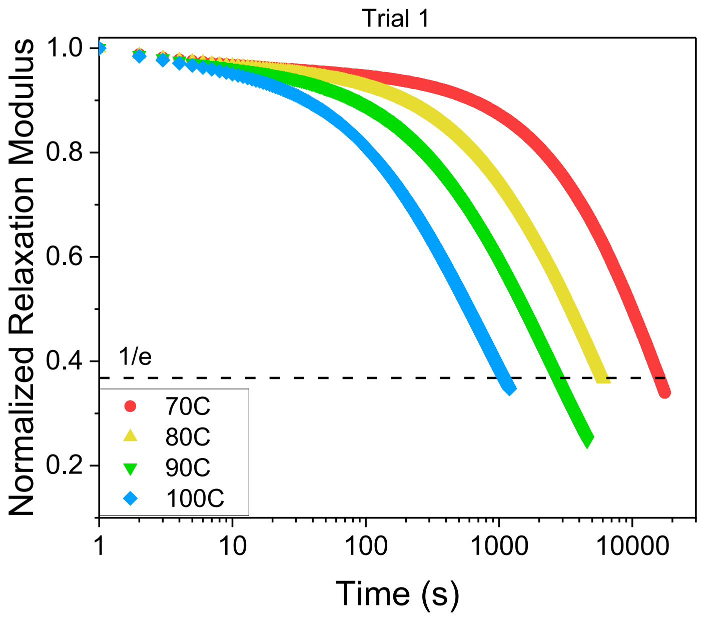

Figure S4. Normalized stress relaxation as a function of time at temperatures ranging from 70 to 100 ºC for **Si-2(50-1)** sample 1.

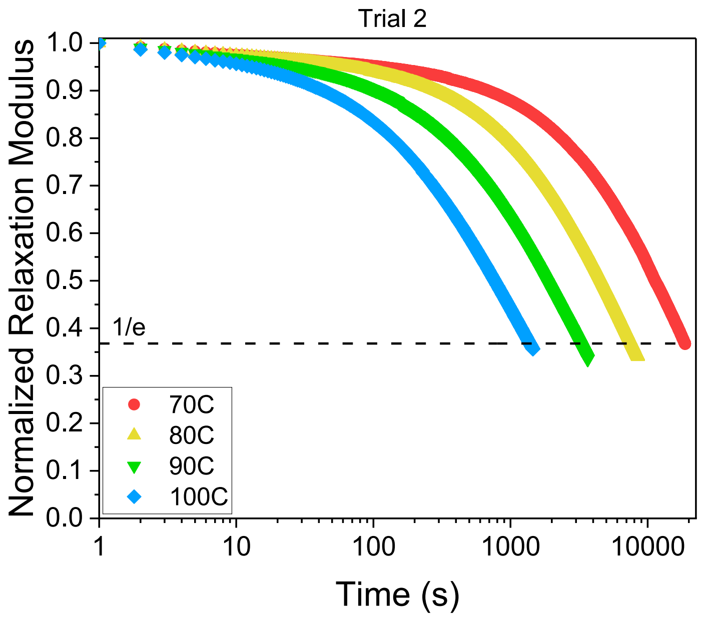

Figure S5. Normalized stress relaxation as a function of time at temperatures ranging from 70 to 100 ºC for **Si-2(50-1)** sample 2.

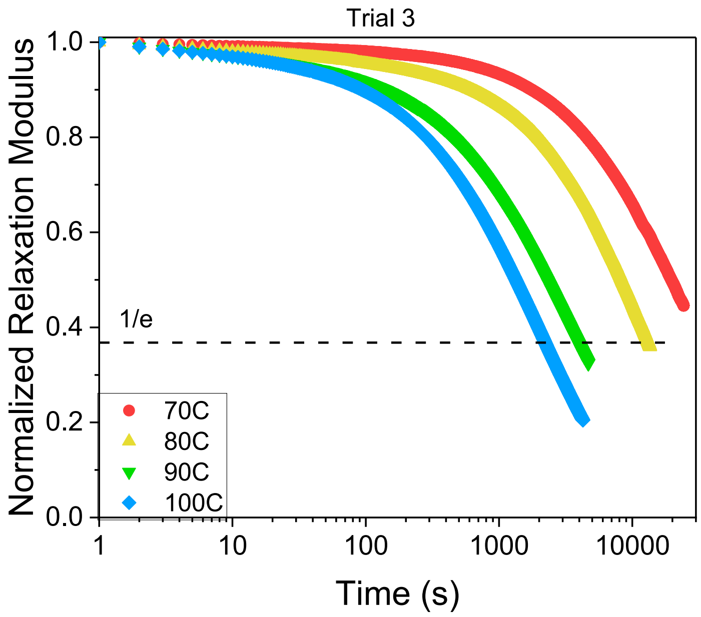

Figure S6. Normalized stress relaxation as a function of time at temperatures ranging from 70 to 100 ºC for **Si-2(50-1)** sample 3.

Table S3. τ values calculated for **Si-2(50-1)** at various temperatures

Temperature (°C)	$\tau_{trial\ 1}$ (s)	$\tau_{trial\ 2}$ (s)	$\tau_{trial\ 3}$ (s)
70	16166	18976	31579
80	6090	7703	13367
90	2691	3187	3904
100	1069	1367	2331

Table S4. β parameters calculated for Si-2(50-1) at various temperatures

Temperature (°C)	$\beta_{trial\ 1}$	$\beta_{trial\ 2}$	$\beta_{trial\ 3}$
70	0.75	0.72	0.77
80	0.68	0.71	0.78
90	0.64	0.66	0.69
100	0.64	0.64	0.72

<u>Si-2(50-2)</u>

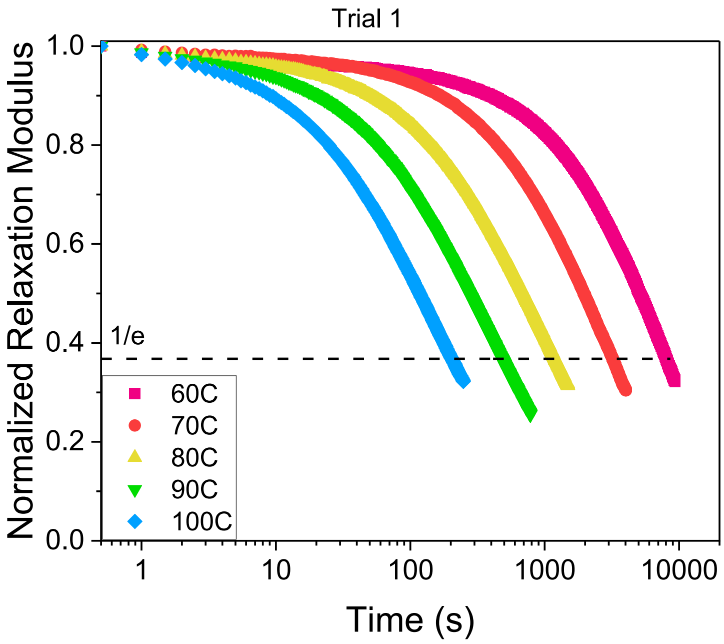

Figure S7. Normalized stress relaxation as a function of time at temperatures ranging from 60 to 100 ºC for **Si-2(50-2)** sample 1.

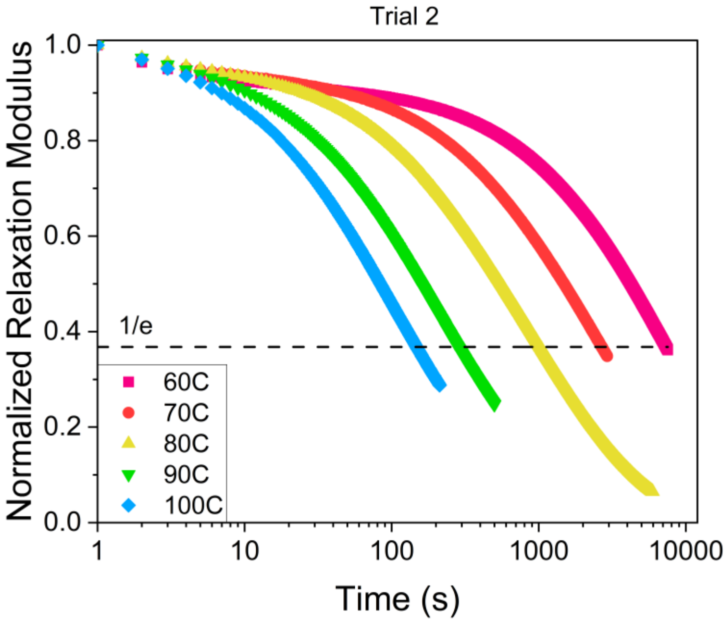

Figure S8. Normalized stress relaxation as a function of time at temperatures ranging from 60 to 100 ºC for **Si-2(50-2)** sample 2.

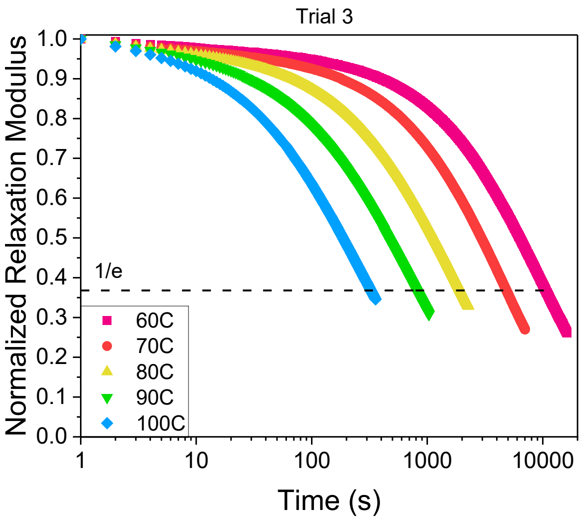

Figure S9. Normalized stress relaxation as a function of time at temperatures ranging from 60 to 100 ºC for **Si-2(50-2)** sample 3.

Table S5. τ values calculated for **Si-2(50-2)** at various temperatures

Temperature (°C)	$\tau_{trial\ 1}$ (s)	$\tau_{trial\ 2}$ (s)	$\tau_{trial\ 3}$ (s)
60	7600	8086	10654
70	2704	3189	4863
80	1045	1190	1943
90	286	482	801
100	148	202	321

Table S6. β values calculated for **Si-2(50-2)** at various temperatures

Temperature (°C)	$\beta_{trial\ 1}$	$\beta_{trial\ 2}$	$\beta_{trial\ 3}$
60	0.61	0.79	0.71
70	0.61	0.76	0.72
80	0.59	0.7	0.69
90	0.65	0.68	0.67
100	0.68	0.7	0.69

Si-2(50-6)

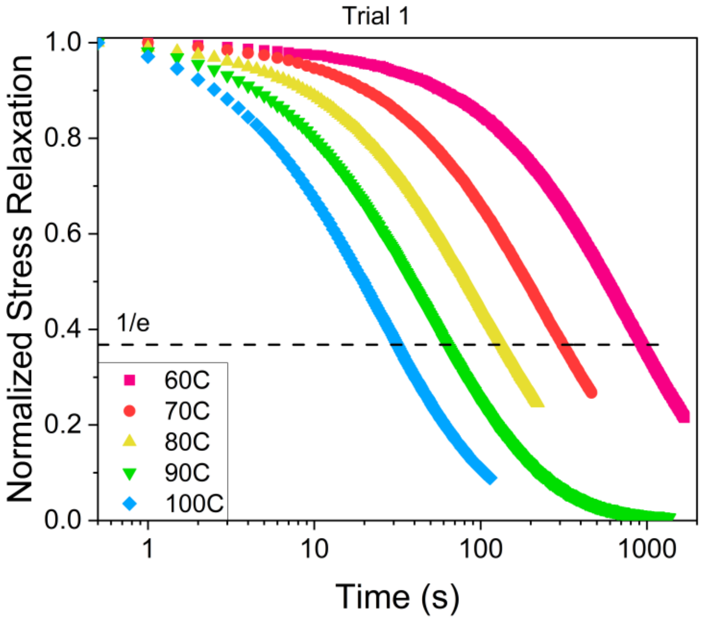

Figure S10. Normalized stress relaxation as a function of time at temperatures ranging from 60 to 100 ºC for **Si-2(50-6)** sample 1.

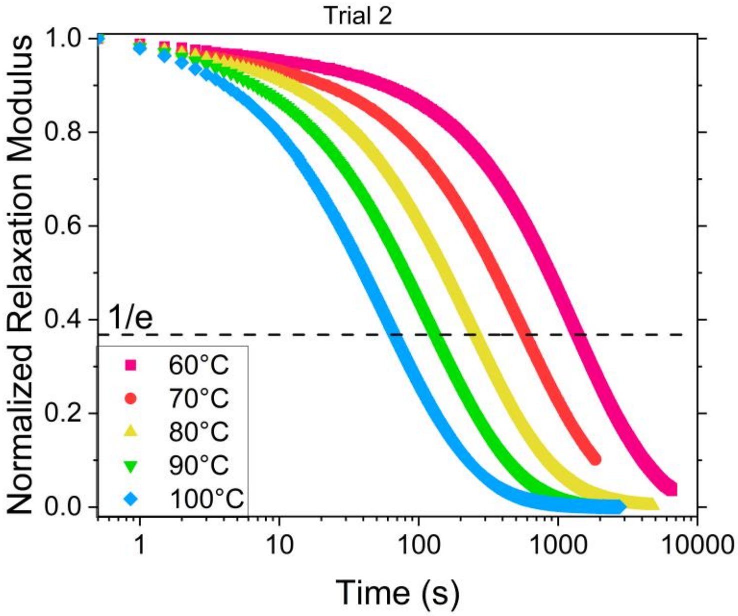

Figure S11. Normalized stress relaxation as a function of time at temperatures ranging from 60 to 100 ºC for **Si-2(50-6)** sample 2.

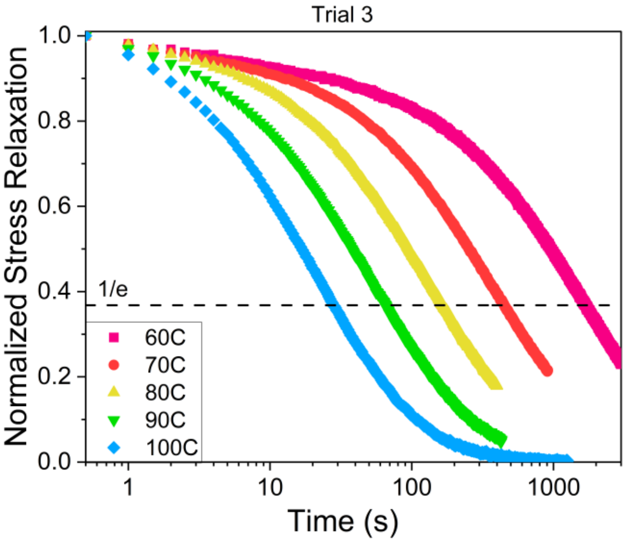

Figure S12. Normalized stress relaxation as a function of time at temperatures ranging from 60 to 100 ºC for **Si-2(50-6)** sample 3.

Table S7. τ values calculated for **Si-2(50-6)** at various temperatures

Temperature (°C)	$\tau_{trial\ 1}$ *(s)*	$\tau_{trial\ 2}$ *(s)*	$\tau_{trial\ 3}$ *(s)*
60	946	1385	1679
70	313	568	454
80	136	267	169
90	65.4	129	67.4
100	32.8	68.5	29.4

Table S8. β values calculated for **Si-2(50-6)** at various temperatures

Temperature (°C)	$\beta_{trial\ 1}$	$\beta_{trial\ 2}$	$\beta_{trial\ 3}$
60	0.78	0.77	0.65
70	0.77	0.73	0.65
80	0.76	0.7	0.63
90	0.67	0.7	0.63
100	0.75	0.7	0.62

<u>Si-2(25-6)</u>

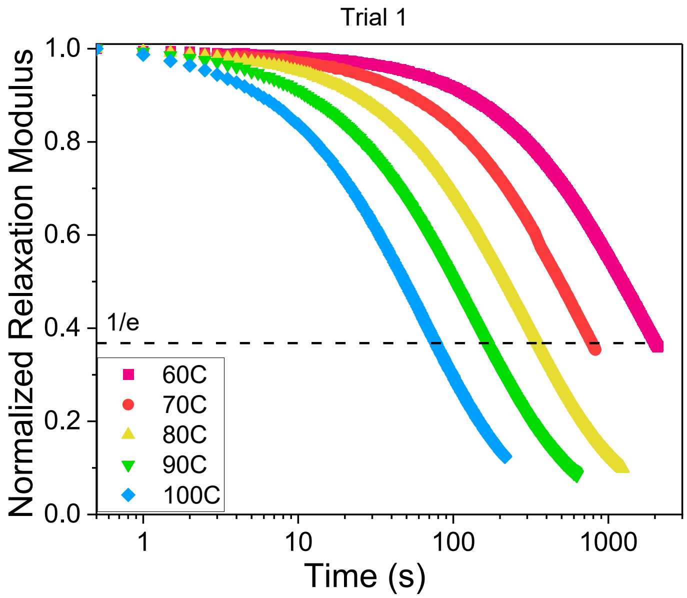

Figure S13. Normalized stress relaxation as a function of time at temperatures ranging from 60 to 100 ºC for **Si-2(25-6)** sample 1.

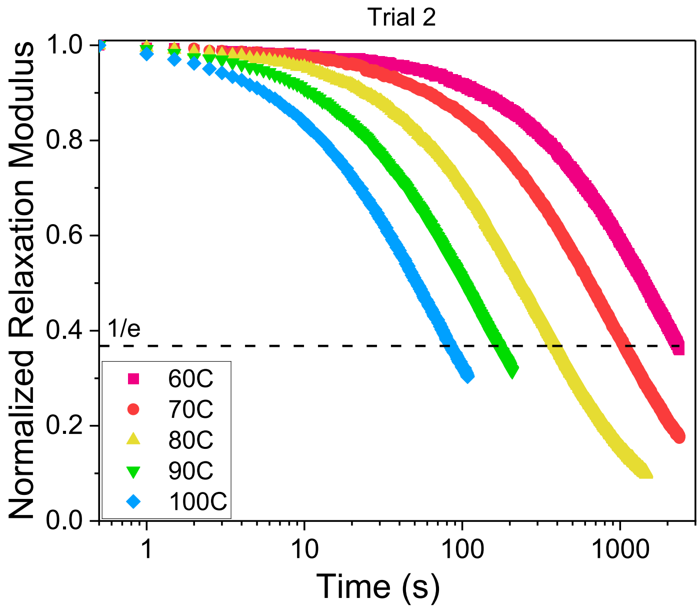

Figure S14. Normalized stress relaxation as a function of time at temperatures ranging from 60 to 100 ºC for **Si-2(25-6)** sample 2.

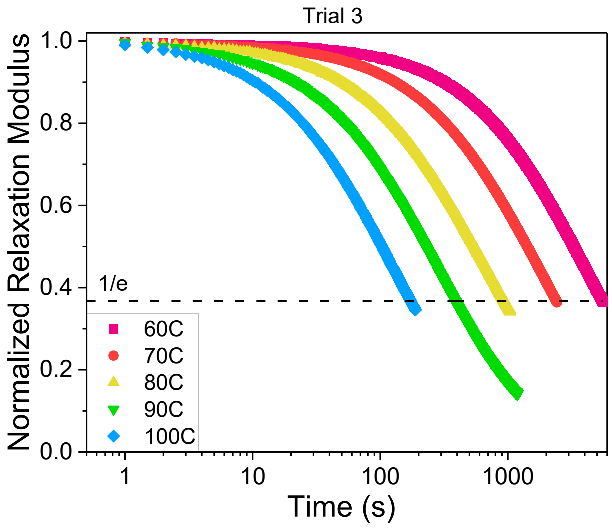

Figure S15. Normalized stress relaxation as a function of time at temperatures ranging from 60 to 100 ºC for **Si-2(25-6)** sample 3.

Table S9. τ values calculated for **Si-2(25-6)** at various temperatures

Temperature (°C)	$\tau_{trial\ 1}$ (s)	$\tau_{trial\ 2}$ (s)	$\tau_{trial\ 3}$ (s)
60	1982	2305	5378
70	771	1082	2343
80	368	408	921
90	169	167	401
100	79.2	84.2	169

Table S10. β values calculated for **Si-2(25-6)** at various temperatures

Temperature (°C)	$\beta_{trial\ 1}$	$\beta_{trial\ 2}$	$\beta_{trial\ 3}$
60	0.78	0.77	0.76
70	0.82	0.74	0.75
80	0.74	0.7	0.73
90	0.74	0.78	0.67
100	0.78	0.78	0.77

Si-2(10-6)

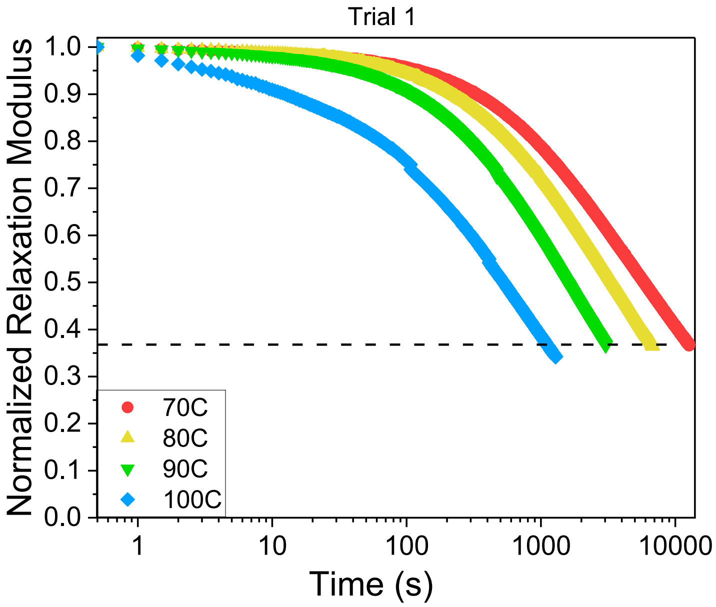

Figure S16. Normalized stress relaxation as a function of time at temperatures ranging from 70 to 100 ºC for **Si-2(10-6)** sample 1.

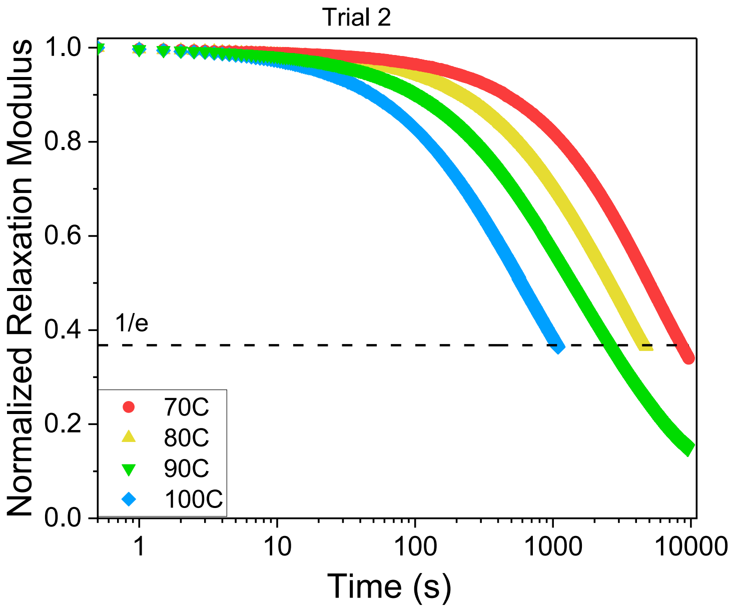

Figure S17. Normalized stress relaxation as a function of time at temperatures ranging from 70 to 100 ºC for **Si-2(10-6)** sample 2.

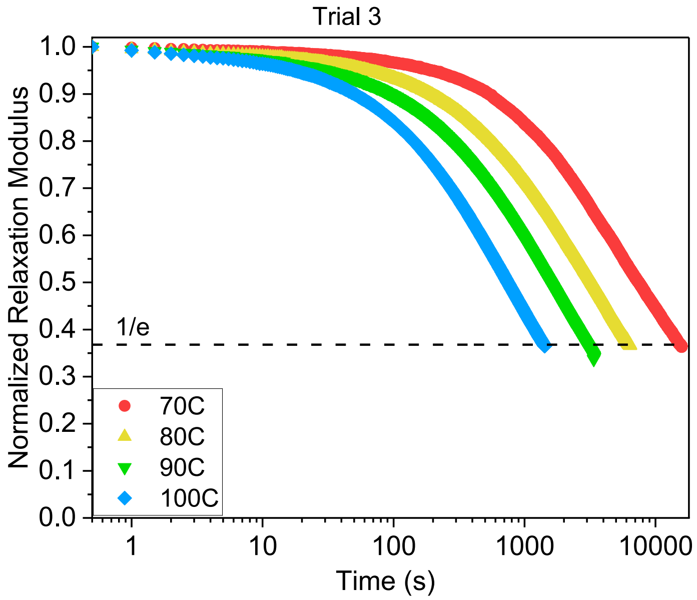

Figure S18. Normalized stress relaxation as a function of time at temperatures ranging from 70 to 100 ºC for **Si-2(10-6)** sample 3.

Table S11. τ values calculated for **Si-2(10-6)** at various temperatures

Temperature (°C)	$\tau_{trial\ 1}$ (s)	$\tau_{trial\ 2}$ (s)	$\tau_{trial\ 3}$ (s)
70	11878	8381	14467
80	6438	4566	6089
90	2875	2674	2918
100	1096	1027	1348

Table S12. β parameters calculated for **Si-2(10-6)** at various temperatures

Temperature (°C)	$\beta_{trial\ 1}$	$\beta_{trial\ 2}$	$\beta_{trial\ 3}$
70	0.55	0.74	0.59
80	0.6	0.71	0.61
90	0.63	0.56	0.62
100	0.51	0.69	0.65

Si-2(60-6)

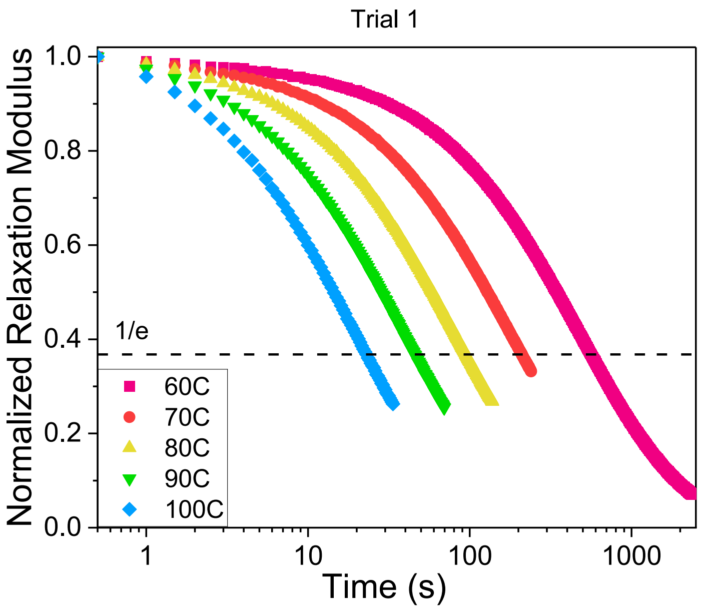

Figure S19. Normalized stress relaxation as a function of time at temperatures ranging from 60 to 100 ºC for **Si-2(60-6)** sample 1.

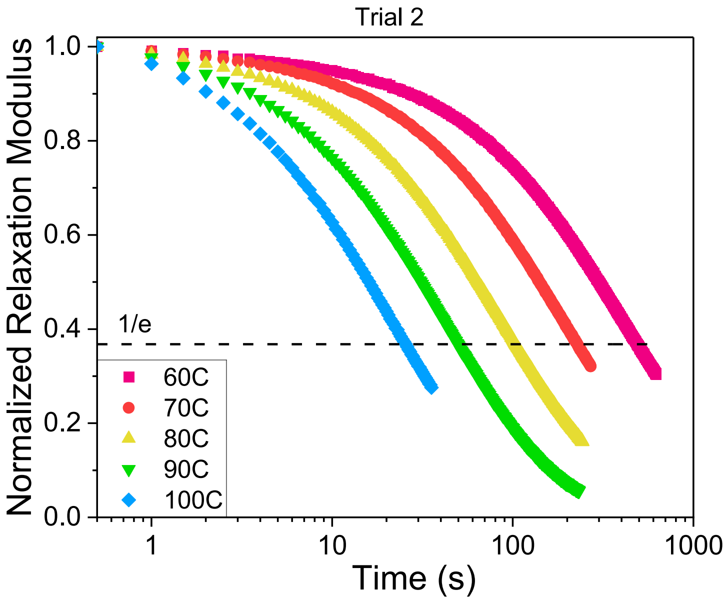

Figure S20. Normalized stress relaxation as a function of time at temperatures ranging from 60 to 100 ºC for **Si-2(60-6)** sample 2.

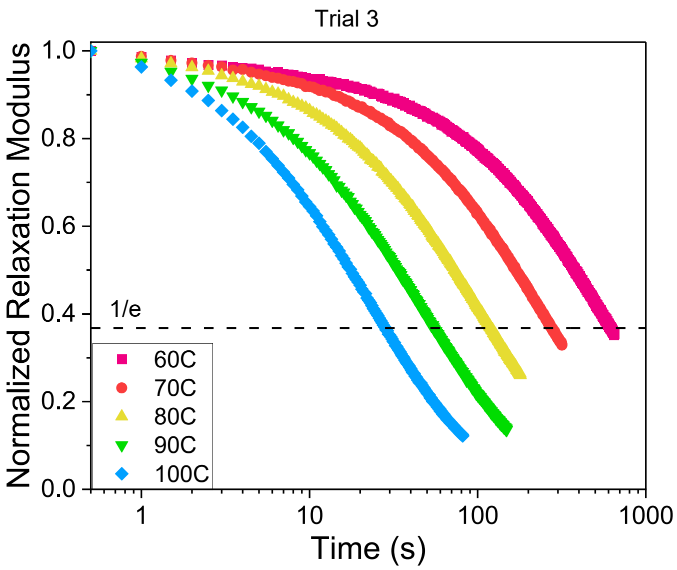

Figure S21. Normalized stress relaxation as a function of time at temperatures ranging from 60 to 100 ºC for **Si-2(60-6)** sample 3.

Table S13. τ values calculated for **Si-2(60-6)** at various temperatures

Temperature (°C)	$\tau_{trial\ 1}$ *(s)*	$\tau_{trial\ 2}$ *(s)*	$\tau_{trial\ 3}$ *(s)*
60	577	487	620
70	208	226	275
80	94.5	106	120
90	45.8	51.2	56
100	23	25.4	29.1

Table S14. β values calculated for **Si-2(60-6)** at various temperatures

Temperature (°C)	$\beta_{trial\ 1}$	$\beta_{trial\ 2}$	$\beta_{trial\ 3}$
60	0.72	0.77	0.74
70	0.78	0.79	0.76
80	0.79	0.76	0.76
90	0.80	0.75	0.74
100	0.84	0.83	0.76

Si-3(50-6)

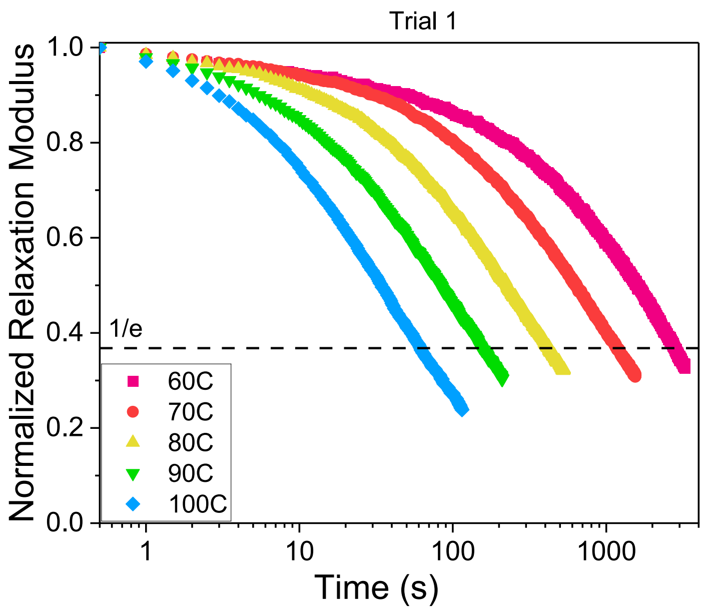

Figure S22. Normalized stress relaxation as a function of time at temperatures ranging from 60 to 100 ºC for **Si-3(50-6)** sample 1.

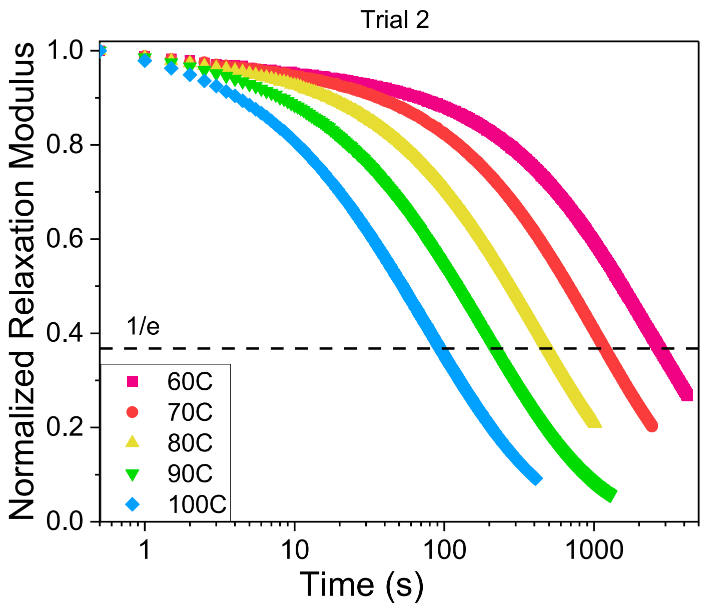

Figure S23. Normalized stress relaxation as a function of time at temperatures ranging from 60 to 100 ºC for **Si-3(50-6)** sample 2.

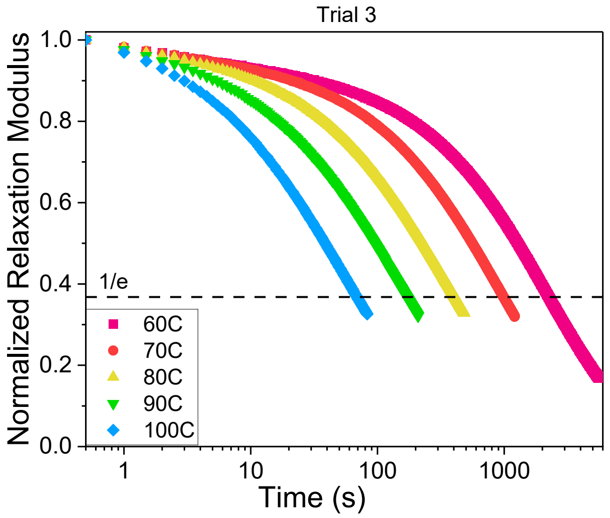

Figure S24. Normalized stress relaxation as a function of time at temperatures ranging from 60 to 100 ºC for **Si-3(50-6)** sample 3.

Table S15. τ values calculated for **Si-3(50-6)** at various temperatures

Temperature (°C)	$\tau_{trial\ 1}$ *(s)*	$\tau_{trial\ 2}$ *(s)*	$\tau_{trial\ 3}$ *(s)*
60	2833	2737	2244
70	1173	1187	995
80	418	502	409
90	154	218	169.5
100	63	96.7	68.1

Table S16. β values calculated for **Si-3(50-6)** at various temperatures

Temperature (°C)	$\beta_{trial\ 1}$	$\beta_{trial\ 2}$	$\beta_{trial\ 3}$
60	0.61	0.64	0.63
70	0.61	0.66	0.63
80	0.62	0.65	0.63
90	0.62	0.62	0.64
100	0.64	0.63	0.67

Si-4(50-6)

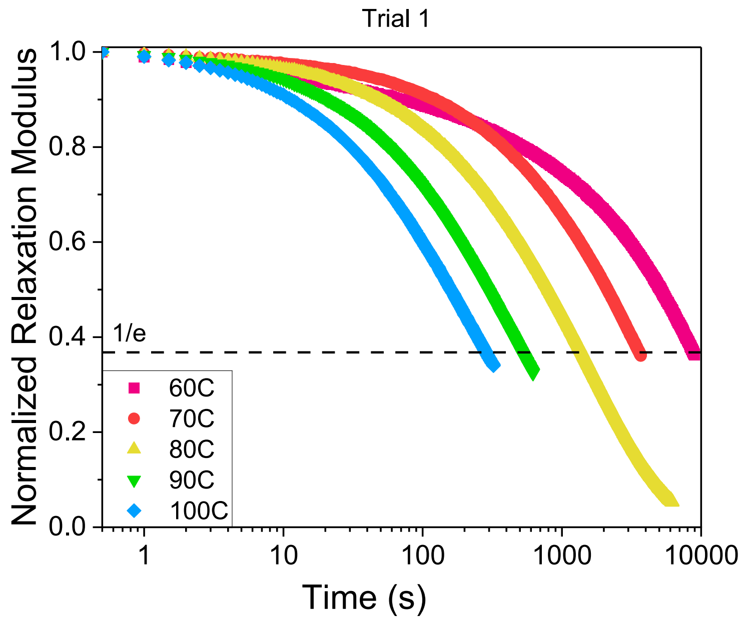

Figure S25. Normalized stress relaxation as a function of time at temperatures ranging from 60 to 100 ºC for **Si-4(50-6)** sample 1.

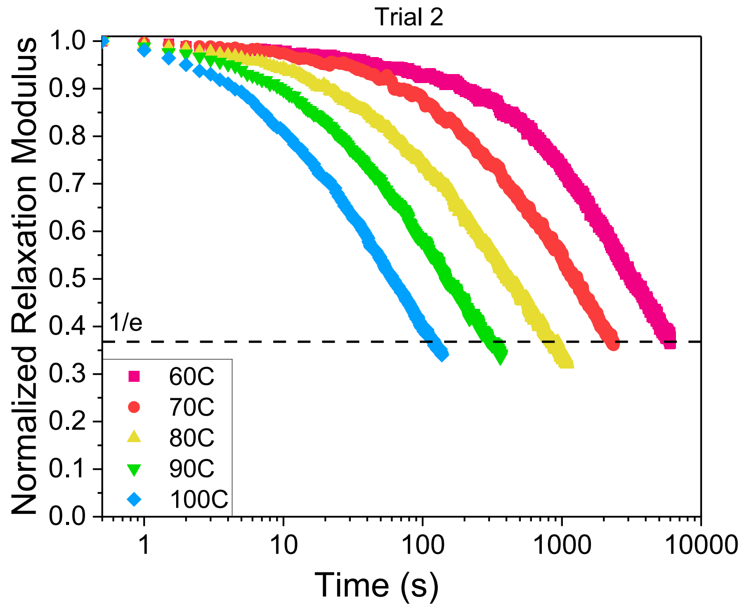

Figure S26. Normalized stress relaxation as a function of time at temperatures ranging from 60 to 100 ºC for **Si-4(50-6)** sample 2.

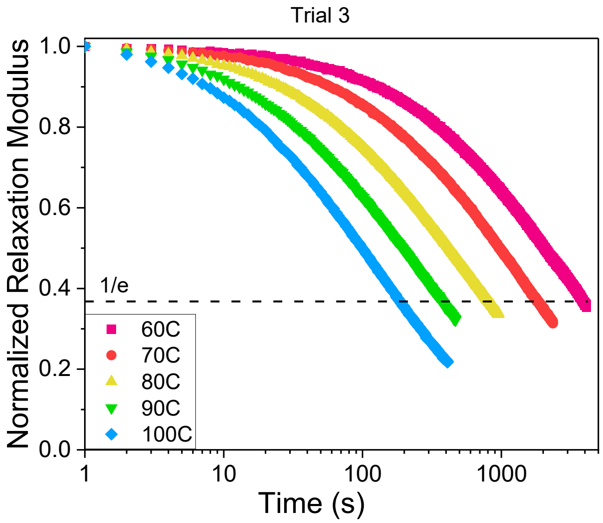

Figure S27. Normalized stress relaxation as a function of time at temperatures ranging from 60 to 100 ºC for **Si-4(50-6)** sample 3.

Table S17. τ values calculated for **Si-4(50-6)** at various temperatures

Temperature (°C)	$\tau_{trial\ 1}$ *(s)*	$\tau_{trial\ 2}$ *(s)*	$\tau_{trial\ 3}$ *(s)*
60	9524	5826	3832
70	3637	2258	1816
80	1370	847	801
90	510	298	365
100	281	115	191

Table S18. β values calculated for **Si-4(50-6)** at various temperatures

Temperature (°C)	$\beta_{trial\ 1}$	$\beta_{trial\ 2}$	$\beta_{trial\ 3}$
60	0.56	0.65	0.61
70	0.67	0.61	0.61
80	0.69	0.56	0.60
90	0.67	0.57	0.60
100	0.66	0.61	0.60

Continuous Relaxation Spectra

The continuous relaxation spectrum for Si-2(50-6) was extracted from the stress relaxation data of trial 2 (Figure 3a in manuscript) between 60 and 100 ºC. The data shows a main mode of relaxation which we attribute to the silyl ether bond exchange.

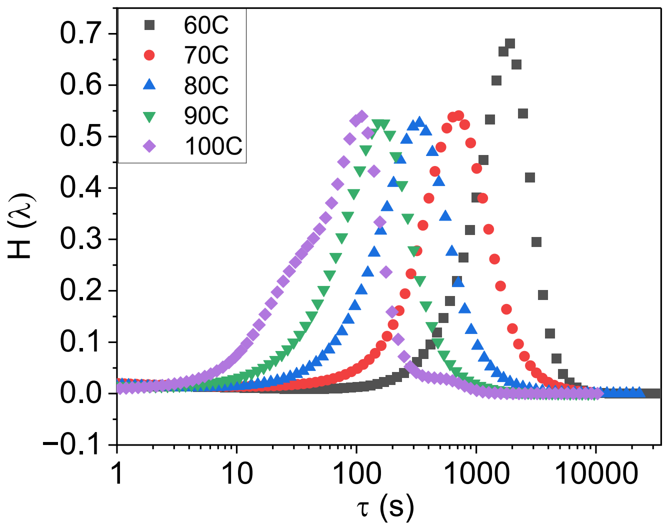

Figure S28. Continuous relaxation spectra for Si-2(50-6) between 60 and 100 ºC

Activation Energy Plots

<u>Varying Cross-linker Length</u>

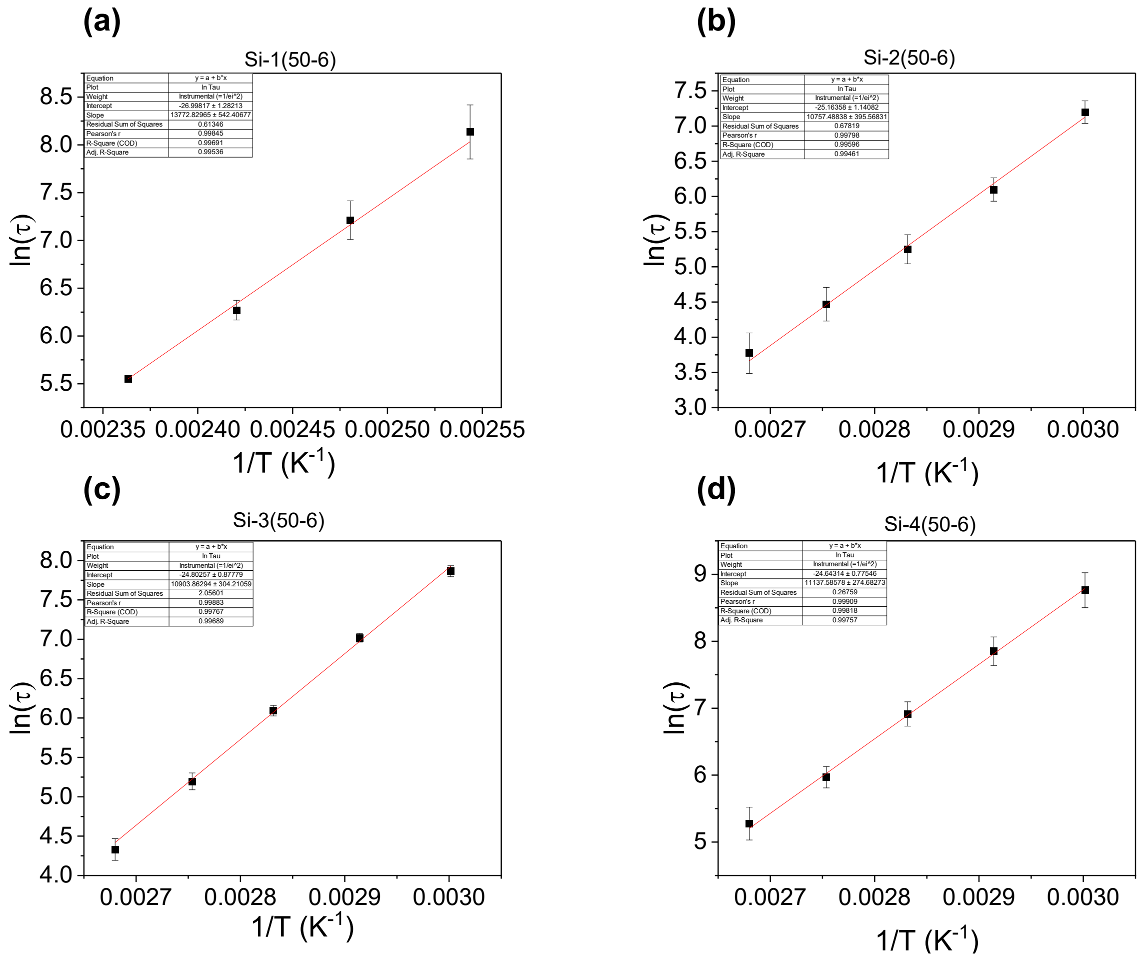

Figure S29. Arrhenius plots for stress relaxation of networks with varying cross-linker lengths. Error bars represent standard error of the mean from triplicates.

Varying wt% pTSA

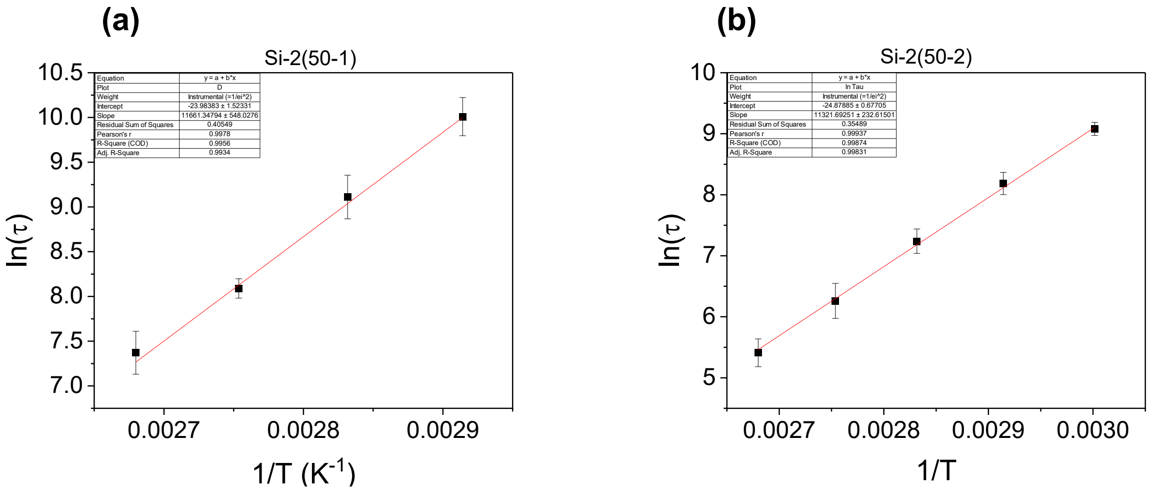

Figure S30. Arrhenius plots for stress relaxation of networks with varying weight percent of pTSA in the network. Error bars represent standard error of the mean from triplicates.

Varying dynamic: static cross-linker ratio

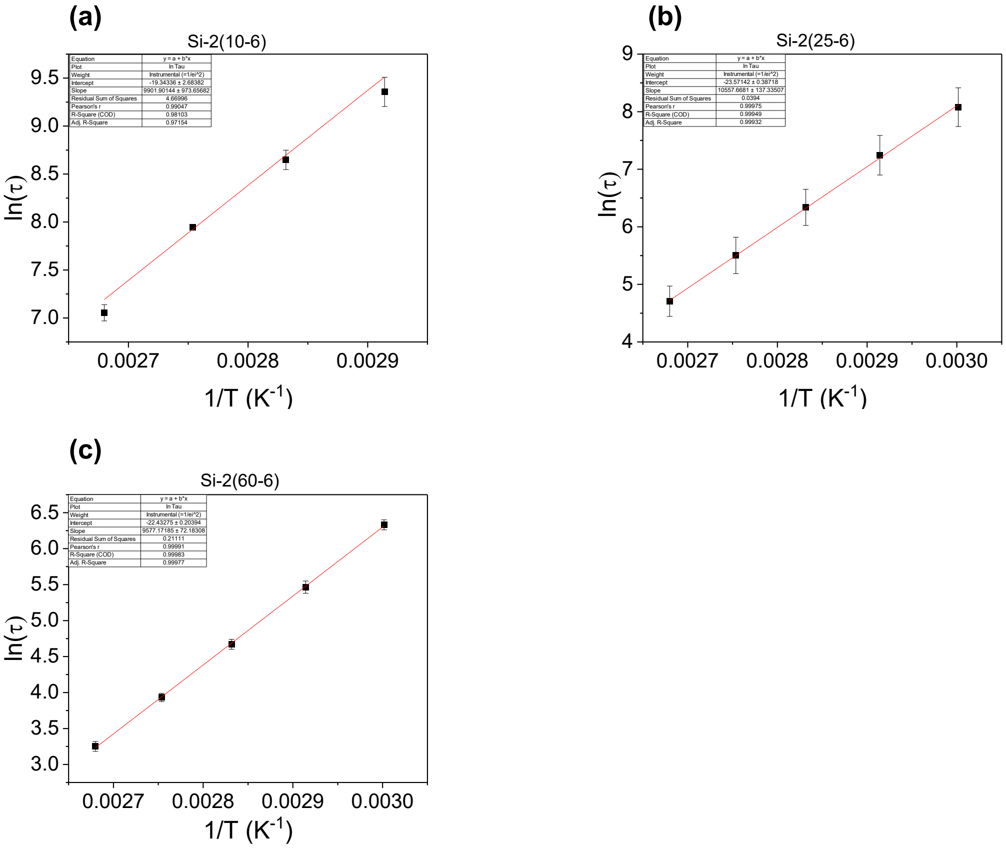

Figure S31. Arrhenius plots for stress relaxation of networks with varying dynamic to static cross-linker in the network. Error bars represent standard error of the mean from triplicates.

NMR Spectra

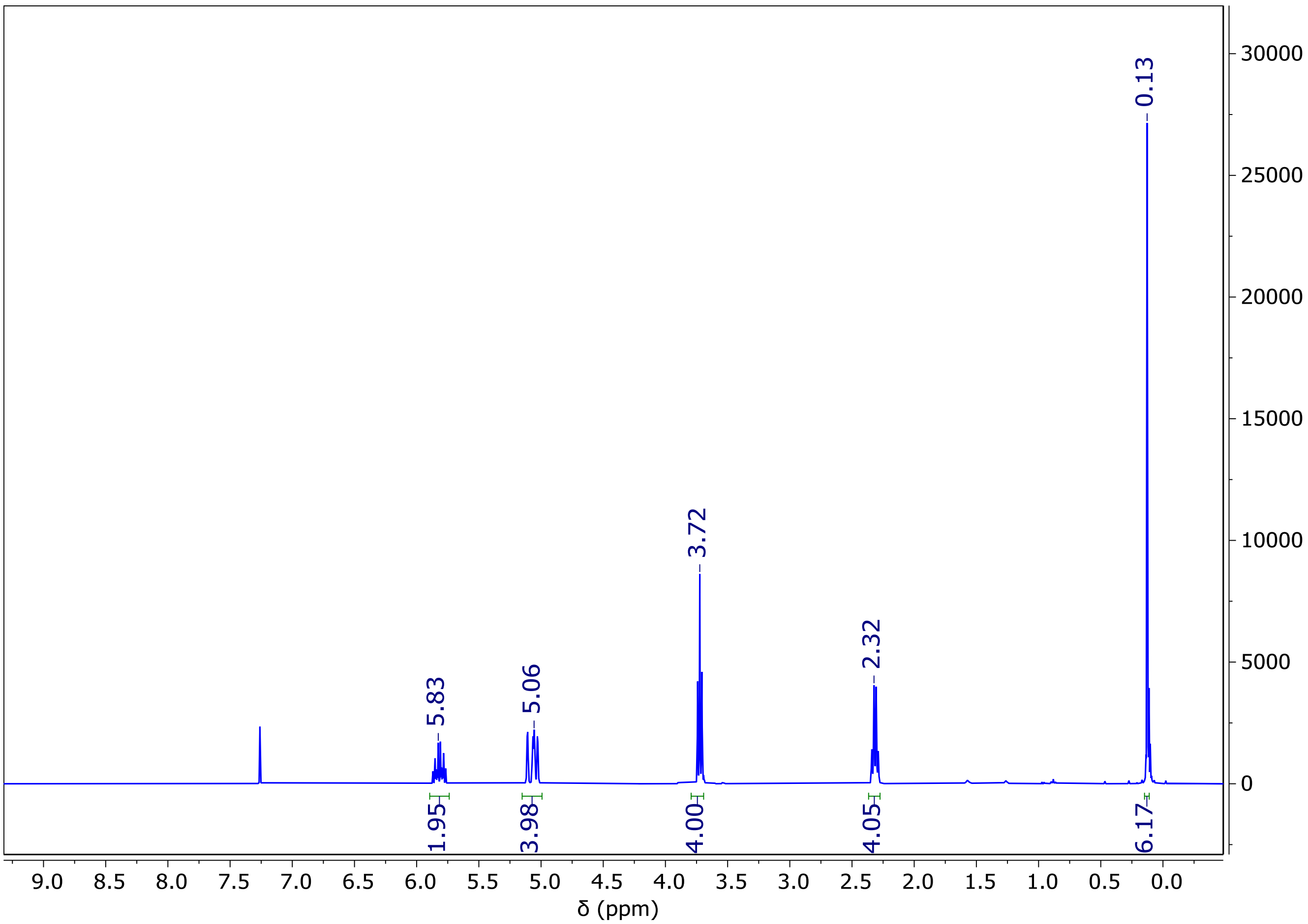

Figure S32.[1] H NMR for Si-1

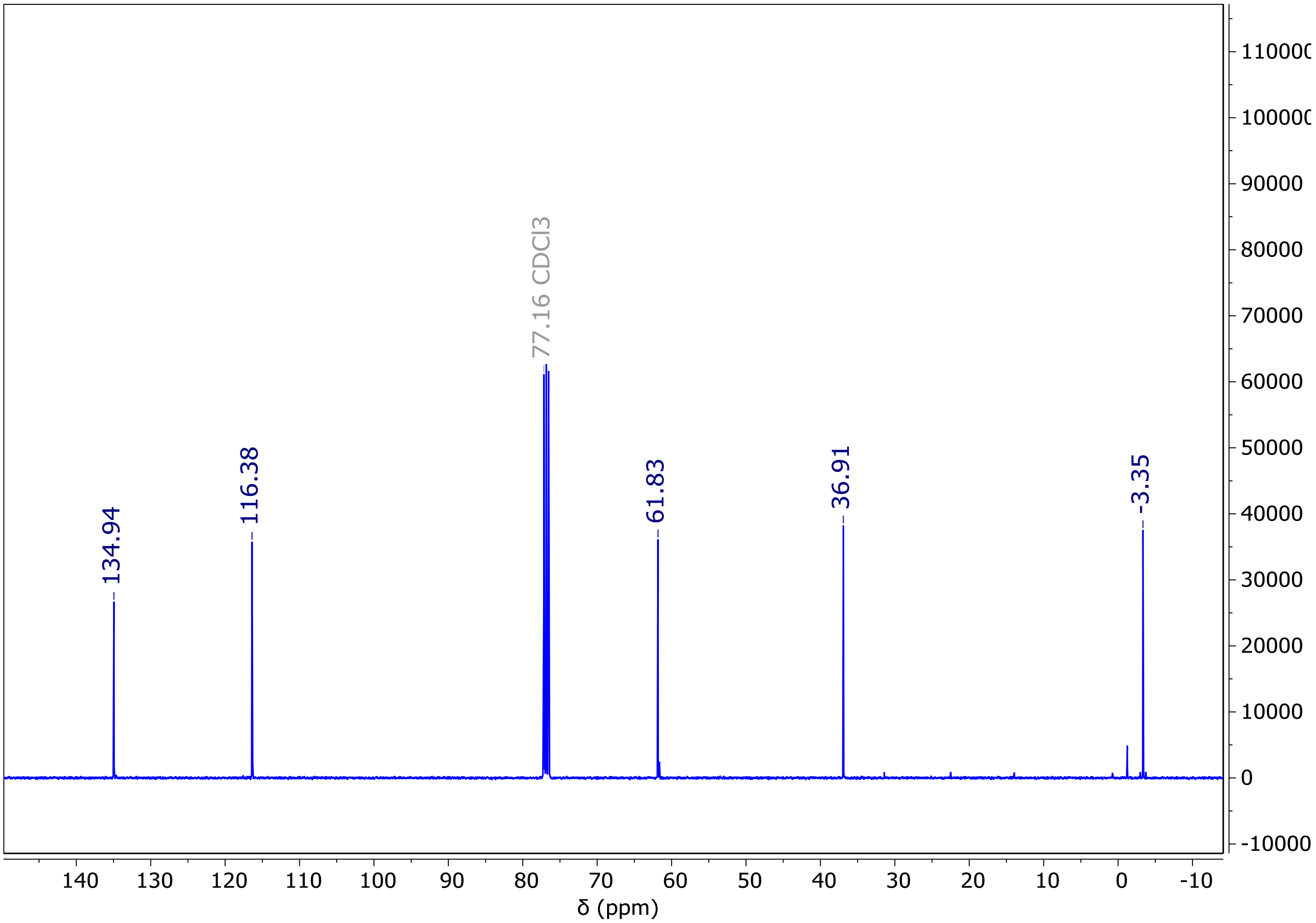

Figure S33..^{13}C NMR for Si-1

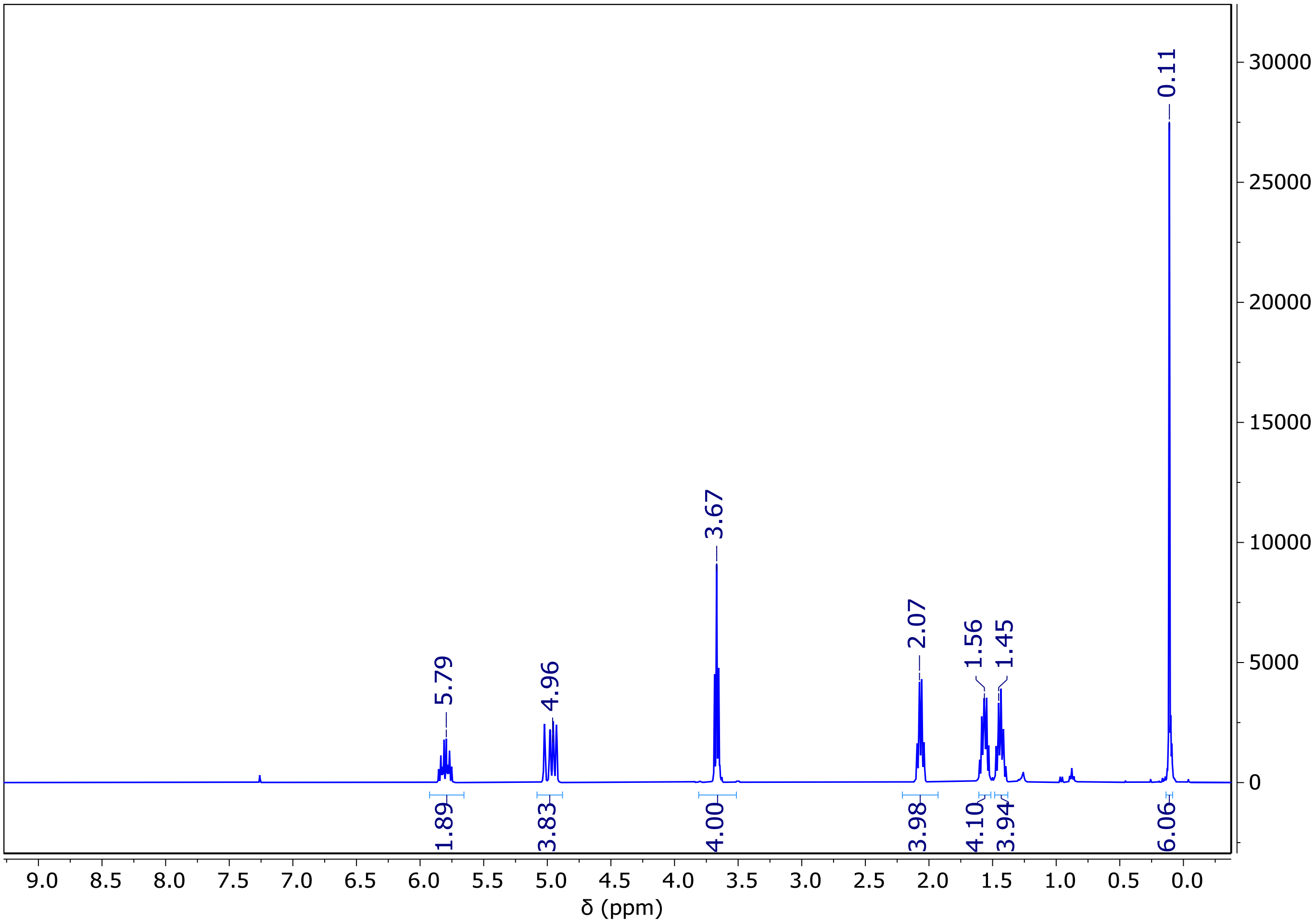

Figure S34.[1] H NMR for Si-2

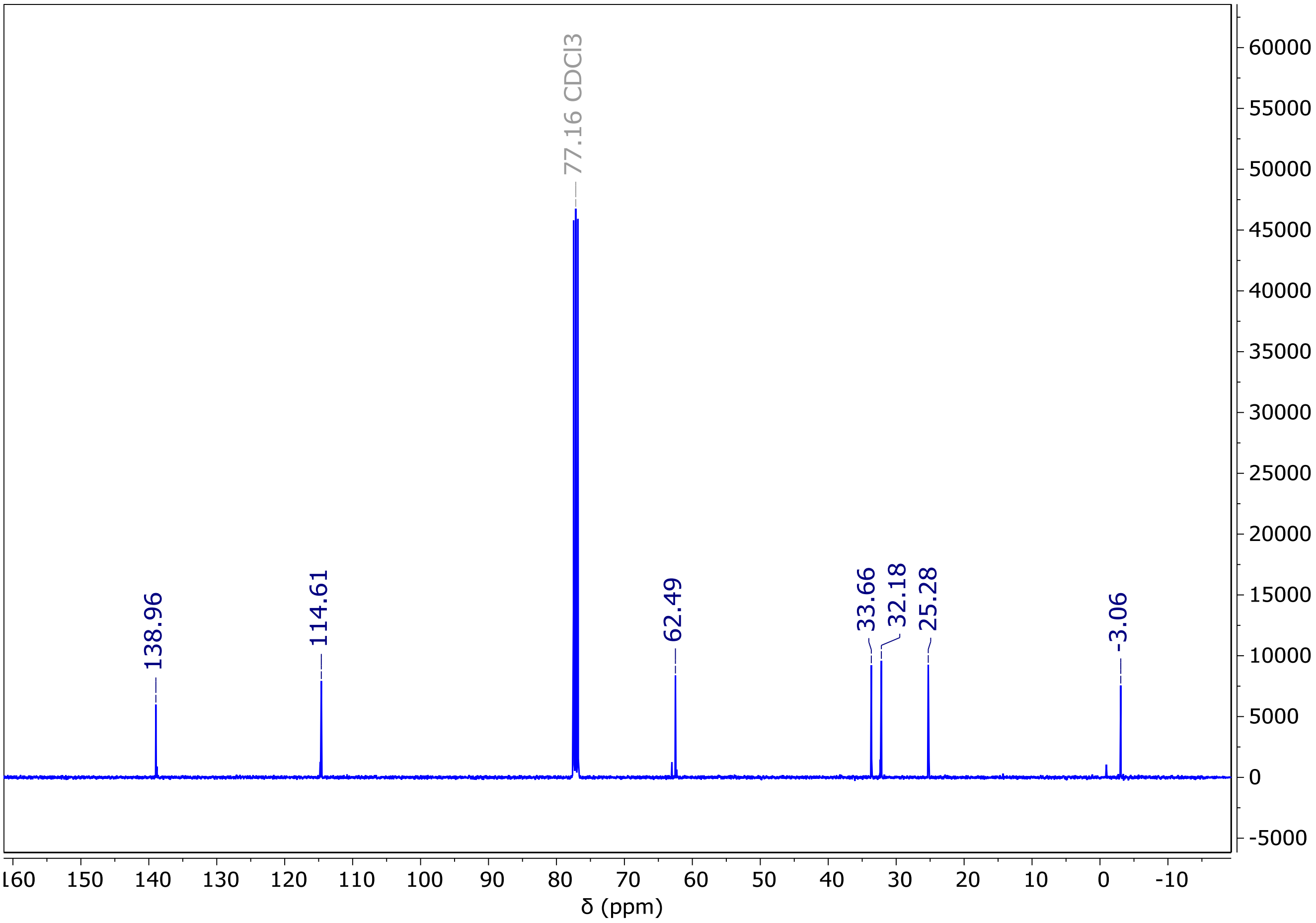

Figure S35. ^{13}C NMR for Si-2

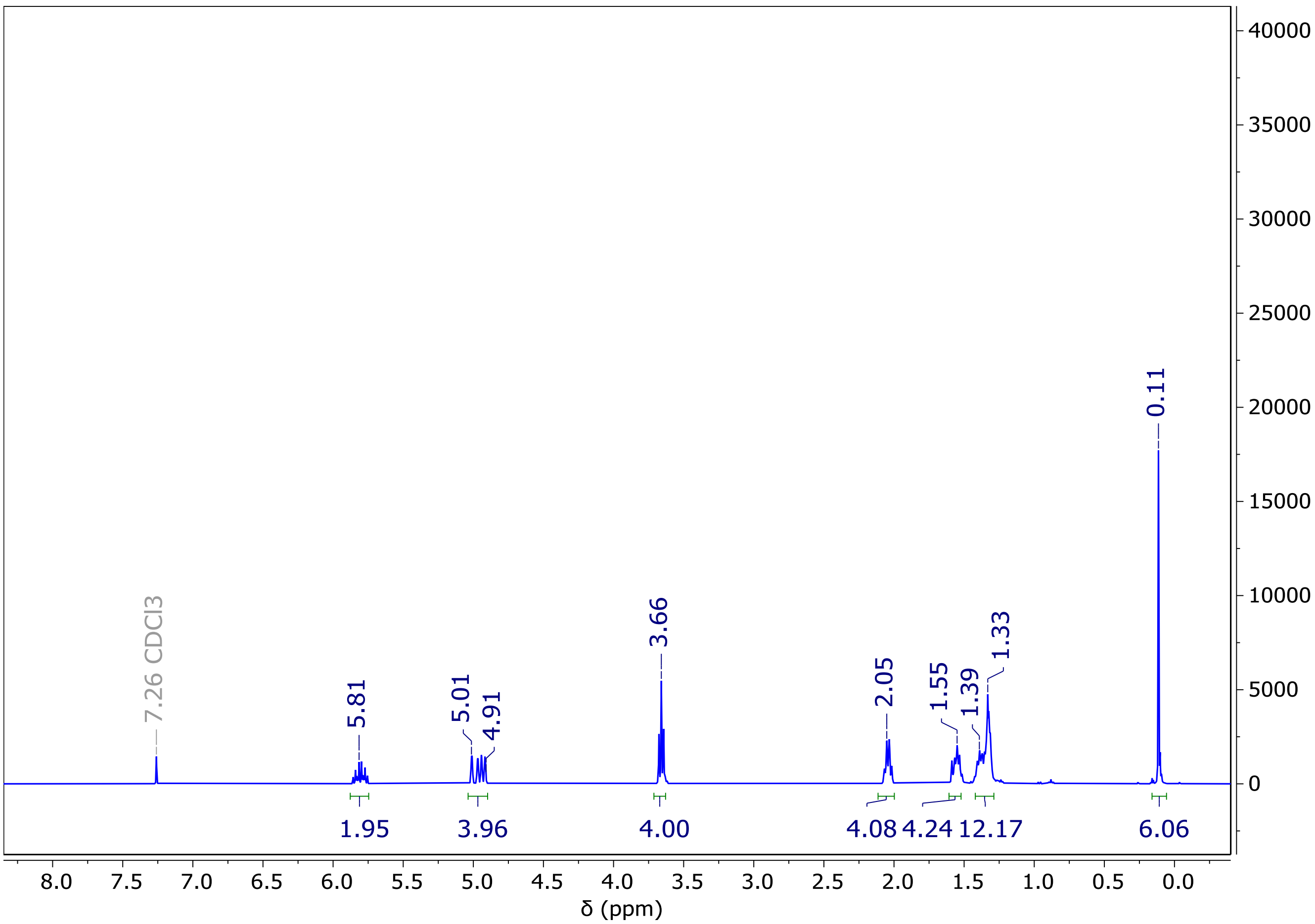

Figure S36.[1] H NMR for Si-3

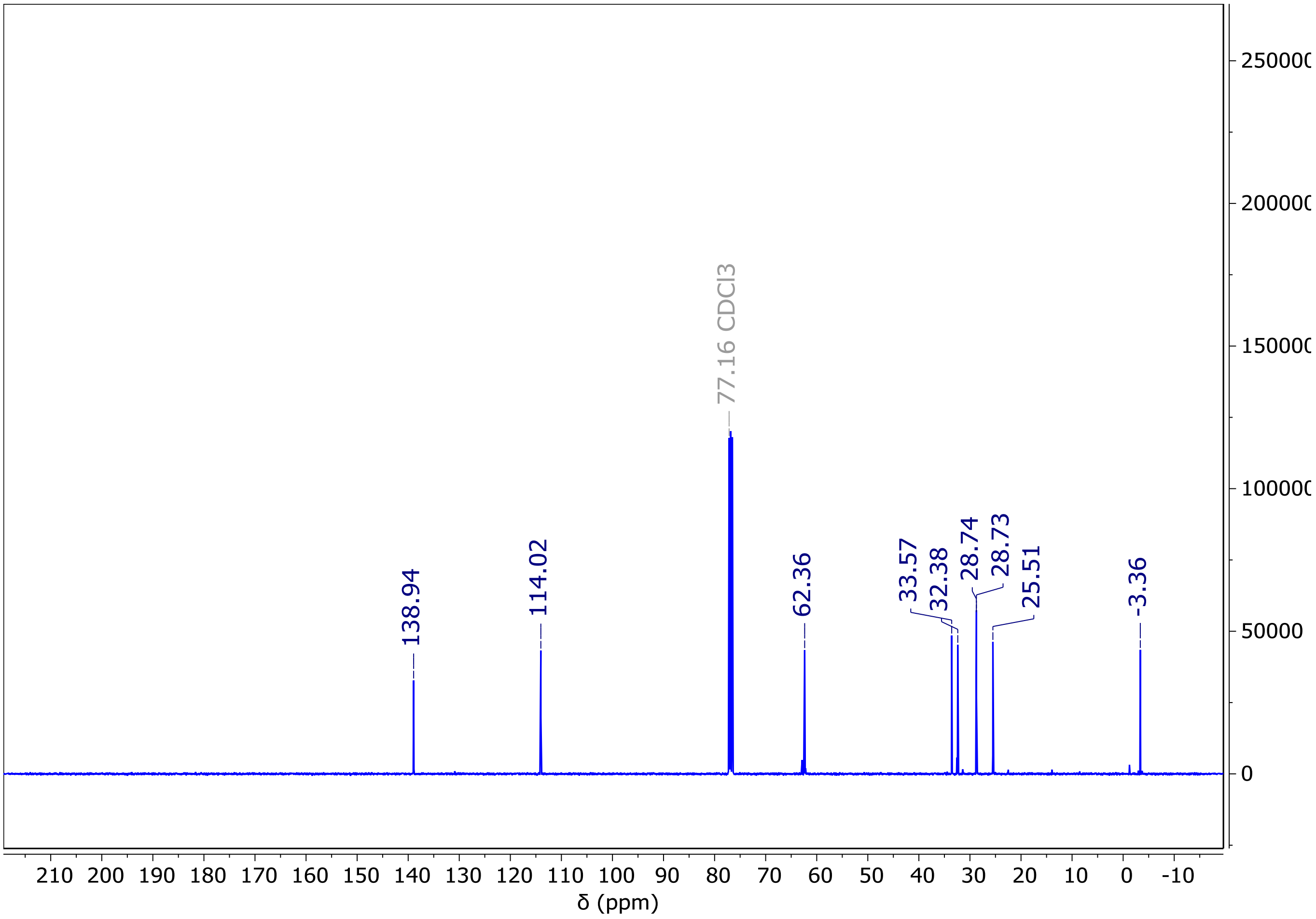

Figure S37.^{13}C NMR for Si-3

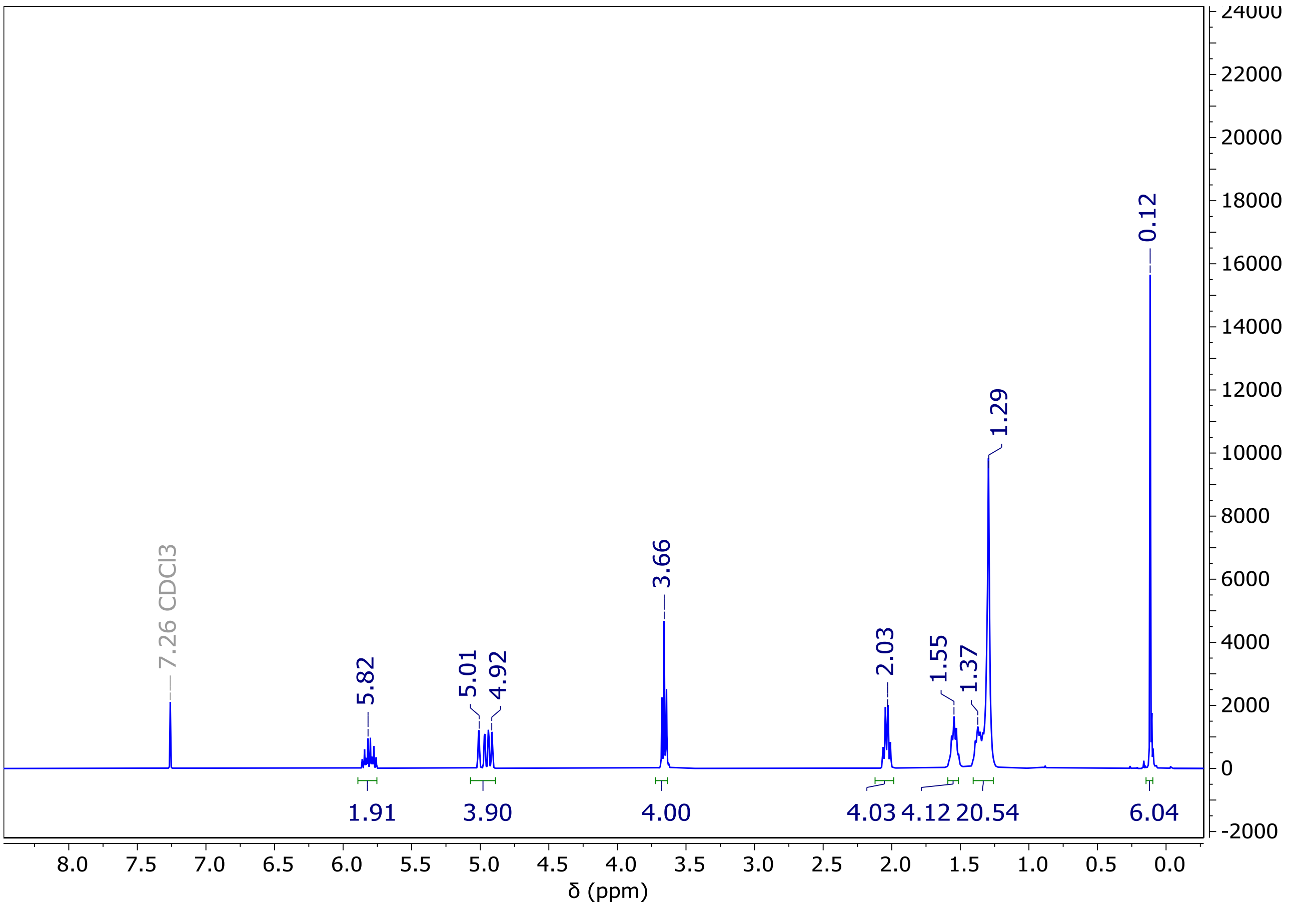

Figure S38.[1] H NMR for Si-4

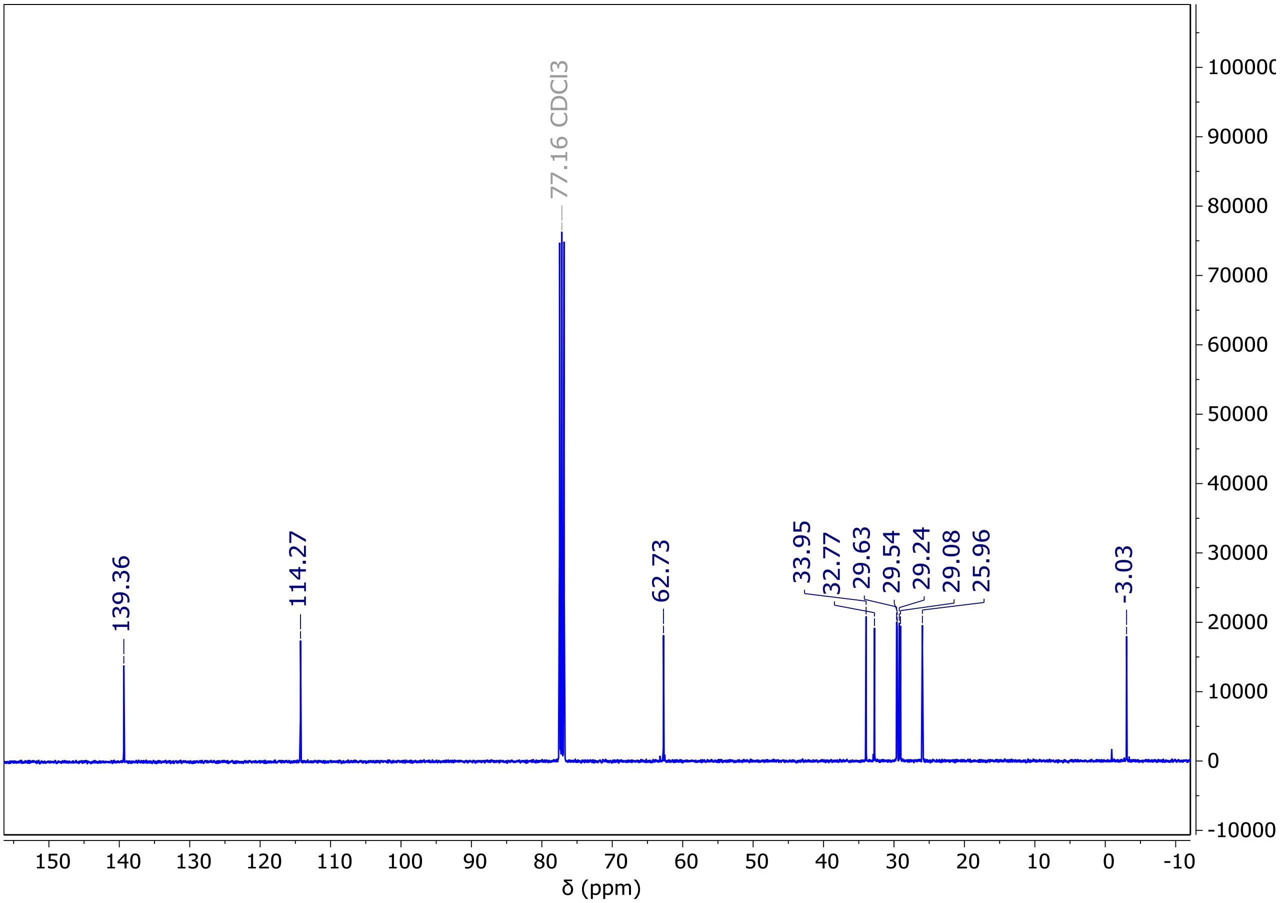

Figure S39.^{13}C NMR for Si-4

Polymer Synthesis

All polymer synthesis followed a similar procedure but varied based on type and quantity of cross-linker in the system as well as amount of pTSA. An example is shown below:

Example: 50% TATATO/50% Si-2 with 6 wt% PTSA

Pentaerythritol tetrakis(3-mercaptopropionate) (PETMP) (300 mg, 0.614 mmol thiol functionality) was combined with crosslinker Si-2 (140 mg, 0.307 mmol alkene functionality, 0.5 equiv.), 1,3,5-triallyl-1,3,5-triazine-2,4,6(1H,3H,5H)-trione (TATATO) (102 mg, 0.307 mmol alkene functionality, 0.5 equiv.), para-toluenesulfonic acid (pTSA) (33 mg, 6 wt%), and photoinitiator 2,2-Dimethoxy-1,2-diphenylethan-1-one (16 mg, 3 wt%). The mixture was mixed thoroughly using a Hauschild 150.1 FVZ-K speedmixer (3500 RPM for 5 min). It was then placed into a 10mm diameter x 1.8mm deep silicone well (Ladd Research Industries). Once in the mold, the samples were cured under 365 nm light using a WheeLED lamp (Mightex) for an hour until the mixture polymerized as a homogeneous, crosslinked thermoset (approximately 1 hour). The samples were then post-cured at 90°C in an oven for 90 minutes to ensure full reactivity.

Polymer Conversion

A Nicolet Nexus 6700 Series Fourier transform infrared (FTIR) spectrometer was used to evaluate the disappearance of the thiol peak and alkene peaks indicating that the polymerization was occurring. The sample was placed in between glass slides, aligned with the laser and a spectrum was taken before and after irradiation of UV (365 nm) light. The area under the curve before and after irradiation was taken to estimate the final conversion of the networks.

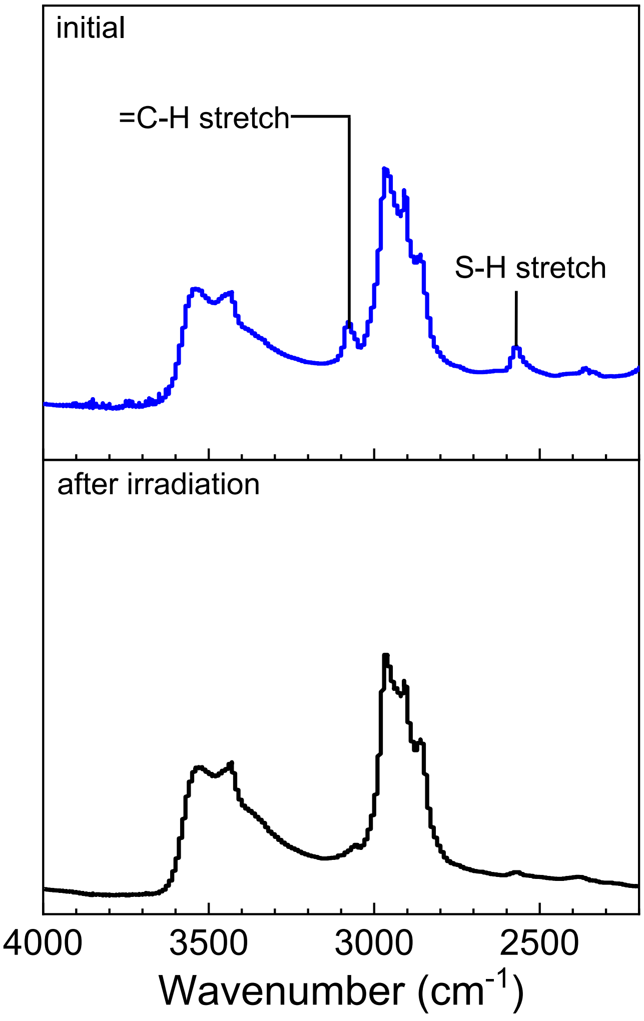

Figure S40. FTIR spectrum showing the window between 2200 and 4000 cm^{-1} highlighting the disappearance of the =C-H and S-H stretches before and after irradiation and heat post-curing for **Si-2(50-6).**

Tg measurements

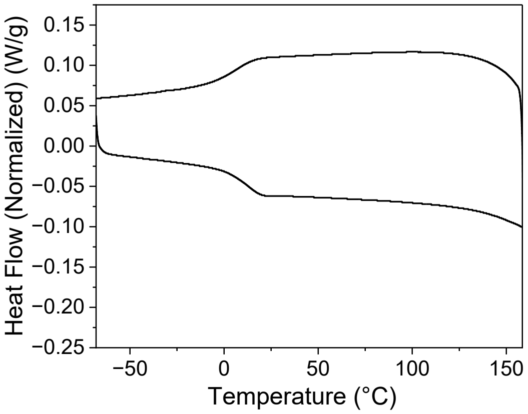

Figure S41. DSC thermogram for **Si-1(50-6)**

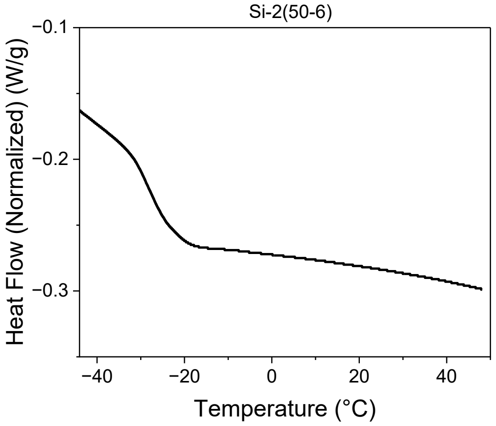

Figure S42. DSC thermogram for **Si-2(50-6)**

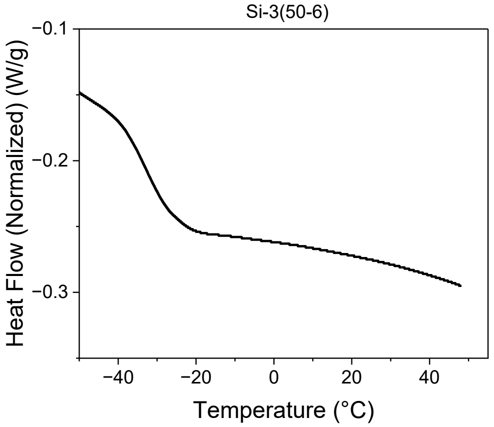

Figure S43. DSC thermogram for **Si-3(50-6)**

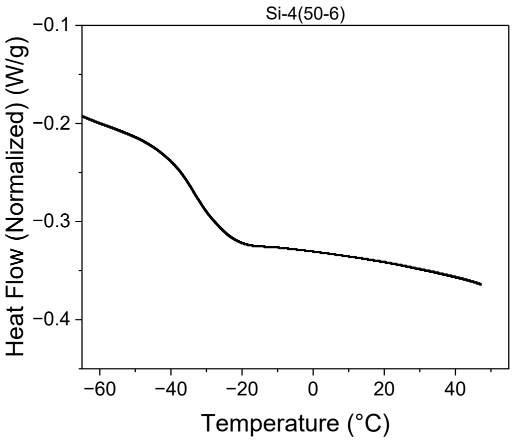

Figure S44. DSC thermogram for **Si-4(50-6)**

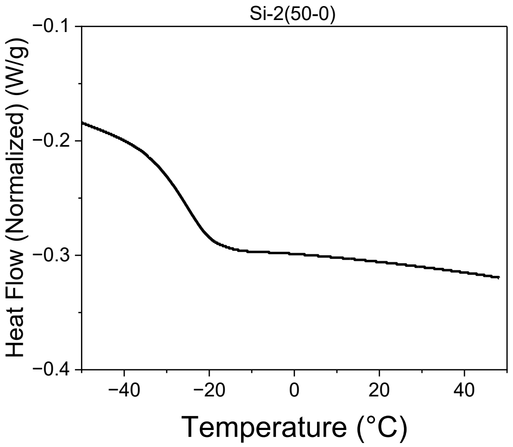

Figure S45. DSC thermogram for **Si-2(50-0)**

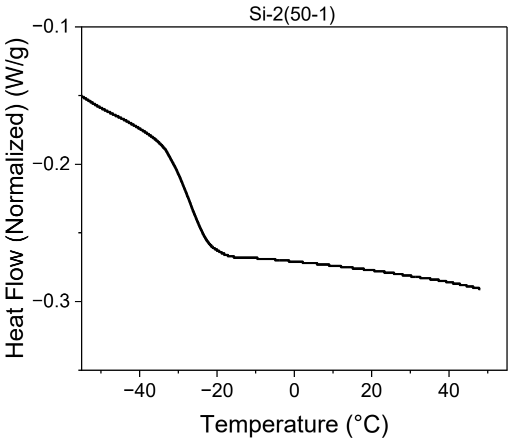

Figure S46. DSC thermogram for **Si-2(50-1)**

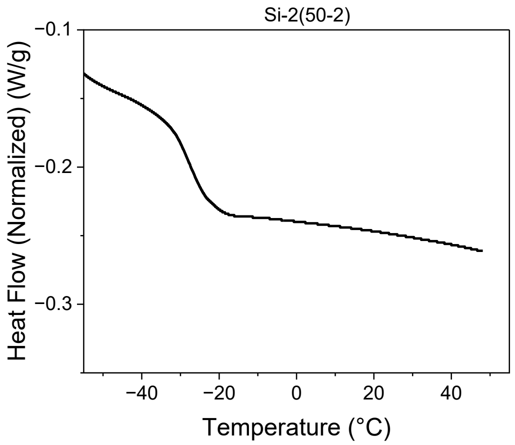

Figure S47. DSC thermogram for **Si-2(50-2)**

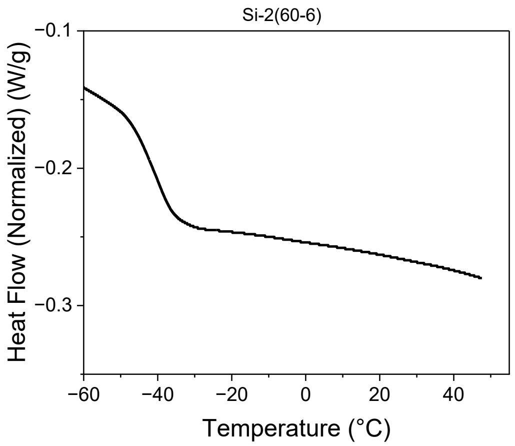

Figure S48. DSC thermogram for **Si-2(60-6)**

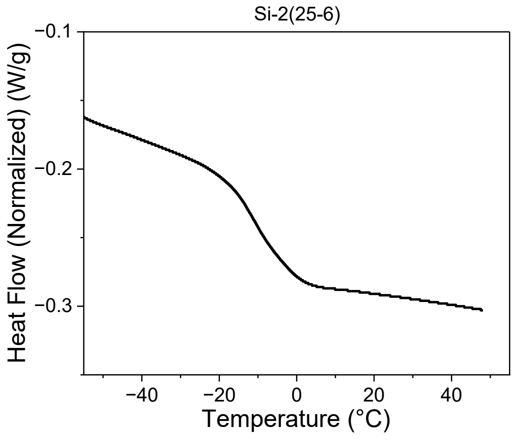

Figure S49. DSC thermogram for **Si-2(25-6)**

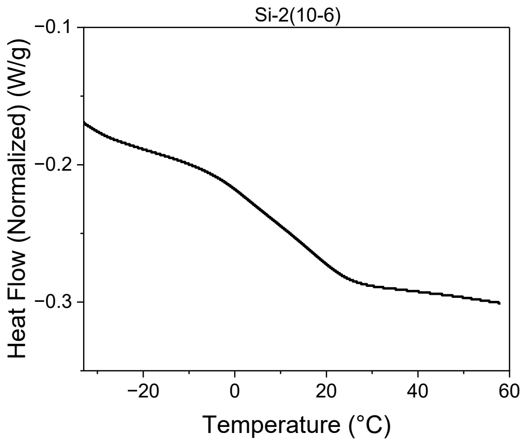

Figure S50. DSC thermogram for **Si-2(10-6)**

Table S19. Glass transition temperature (Tg,onset; Tg,midpoint, and Tg,endpoint) for various synthesized polymer networks

Sample	Tg onset (˚C)	Tg midpoint (˚C)	Tg endpoint (˚C)
**Si-1(50-6)**	2.48	10.77	19.18
**Si-2(50-6)**	-31.97	-27.15	-22.35
**Si-3(50-6)**	-38.51	-32.20	-25.88
**Si-4(50-6)**	-39.60	-32.21	-24.86
**Si-2(50-0)**	-32.97	-25.95	-18.93
**Si-2(50-1)**	-32.82	-27.23	-21.67
**Si-2(50-2)**	-30.95	-26.66	-22.44
**Si-2(60-6)**	-46.67	-40.88	-35.06
**Si-2(25-6)**	-16.69	-9.28	-1.82
**Si-2(10-6)**	-0.26	11.48	23.38

Frequency Sweep Data

Frequency sweeps were performed at constant strain (1%) and at an angular frequency between 10 and 100 rad/s.

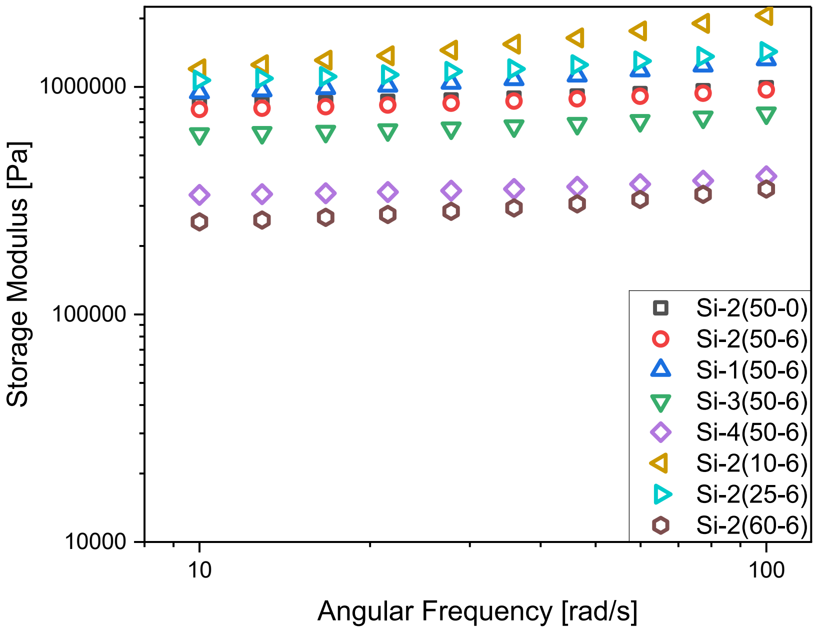

.

Figure S51. Frequency sweep data for various synthesized polymer networks

Diallyl Carbonate Stress Relaxation Data

In these studies, diallyl carbonate (**DAC**) replaced **TATATO** as the co-crosslinker. A 50/50 mol% **Si-1** or **Si-2/DAC** was utilized with PETMP. The stress relaxation data is shown below at 100 ˚C:

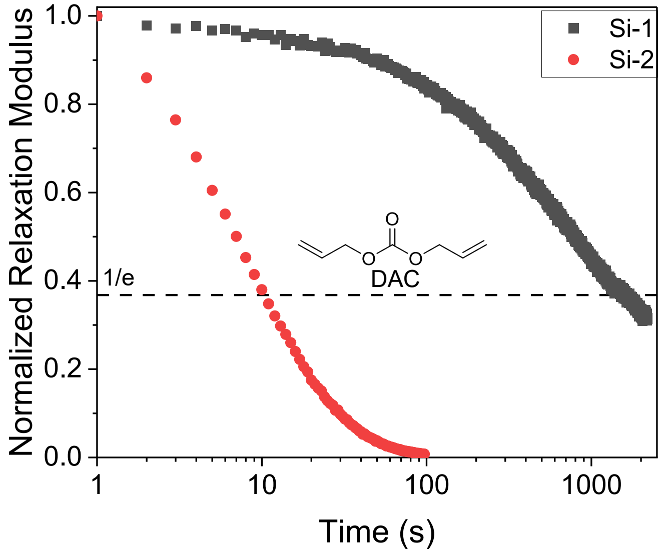

Figure S52. Normalized stress relaxation data for a 50/50 mol% Si-1/DAC and Si-2/DAC sample at 100ºC.

Small molecule kinetics

Procedure

A 1.5mol% solution of pTSA in deuterated benzene was prepared and stirred for an hour to ensure full dissolution of the catalyst. 100 mM of either **Si-1**, **Si-2** or **Si-3** were added to 0.5 mL aliquots of the stock solution. Before the start of each experiment, dimethoxydimethylsilane was added in an equimolar amount, starting the reaction. The contents were quickly transferred to an NMR tube and the measurements began at ~5 minute increments. Reaction conversion was evaluated using the integration of the three peaks between 0.00 and 0.20 ppm which correspond to the hydrogens on the silicon methyl groups. Specifically, the intermediate peak (product peak) was integrated at 1.00 and conversion was calculated using the following equation:

$$\frac{Product\ Peak\ Integration}{\sum Reactant\ and\ Product\ Peak\ Integrations}$$

Si-1 Kinetics:

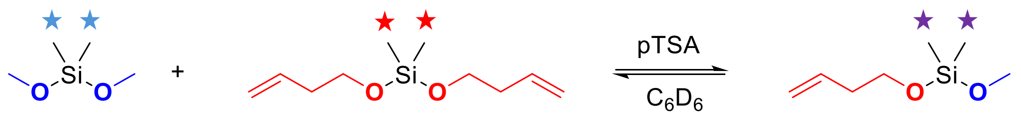

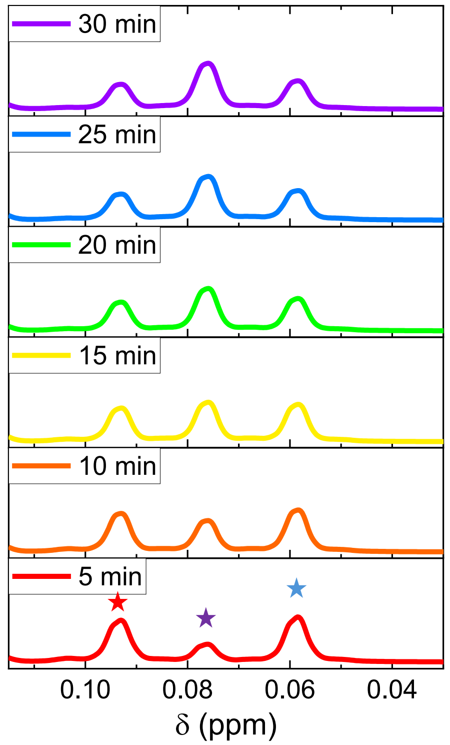

Figure S53. ^{1}HNMR spectrum of the reaction between Si-1 and dimethyoxydimethylsilane between 0.02 and 0.13 ppm as a function of time.

Table S20. Reactant and product integrations at different times of the reaction and subsequent calculated conversion for reaction shown in Figure S50.

Time (min)	Integration Reactant 1	Integration Product	Integration Reactant 2	Conversion
**5**	2.33	1.00	2.44	**0.17**
**10**	1.33	1.00	1.46	**0.26**
**15**	0.84	1.00	0.90	**0.36**
**20**	0.66	1.00	0.70	**0.42**
**25**	0.59	1.00	0.64	**0.45**
**30**	0.53	1.00	0.58	**0.47**

Si-2 Kinetics:

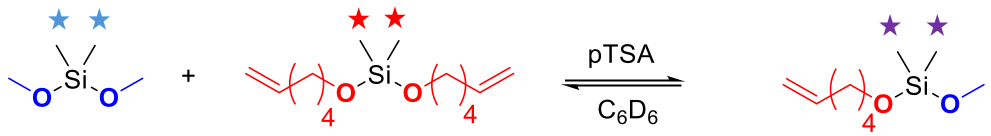

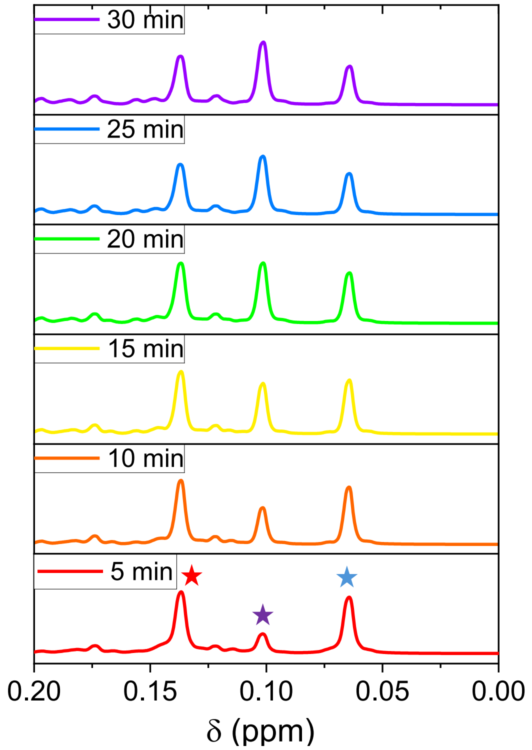

Figure S54. ^{1}HNMR spectrum of the reaction between **Si-2** and dimethyoxydimethylsilane between 0.00 and 0.20 ppm as a function of time.

Table S21. Reactant and product integrations at different times of the reaction and subsequent calculated conversion for reaction shown in Figure S51.

Time (min)	Integration Reactant 1	Integration Product	Integration Reactant 2	Conversion
**5**	2.96	1	2.97	**0.14**
**10**	1.68	1	1.55	**0.24**
**15**	1.25	1	1.07	**0.30**
**20**	1.01	1	0.81	**0.35**
**25**	0.86	1	0.67	**0.40**
**30**	0.75	1	0.55	**0.43**

Si-3 Kinetics:

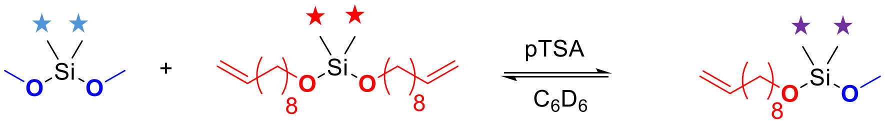

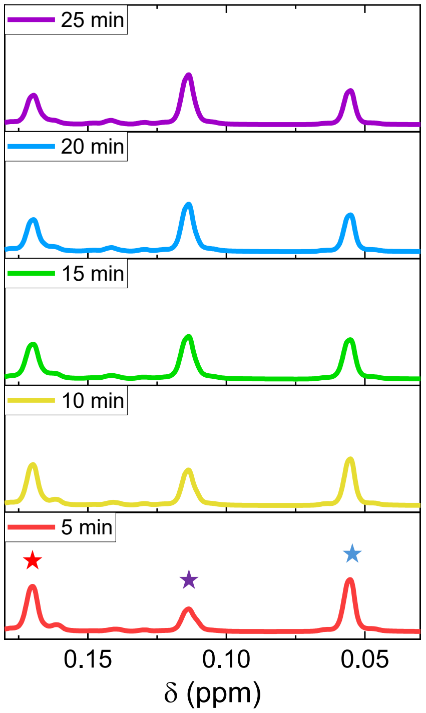

Figure S53. ^{1}HNMR spectrum of the reaction between **Si-3** and dimethyoxydimethylsilane between 0.02 and 0.18 ppm as a function of time.

Table S22. Reactant and product integrations at different times of the reaction and subsequent calculated conversion for reaction shown in Figure S52.

Time (min)	Integration Reactant 1	Integration Product	Integration Reactant 2	Conversion
**5**	1.9	1	1.94	**0.21**
**10**	1.12	1	1.13	**0.31**
**15**	0.88	1	0.85	**0.37**
**20**	0.66	1	0.73	**0.42**
**25**	0.61	1	0.59	**0.45**

Reprocessed Samples Data

Stress-Strain Data

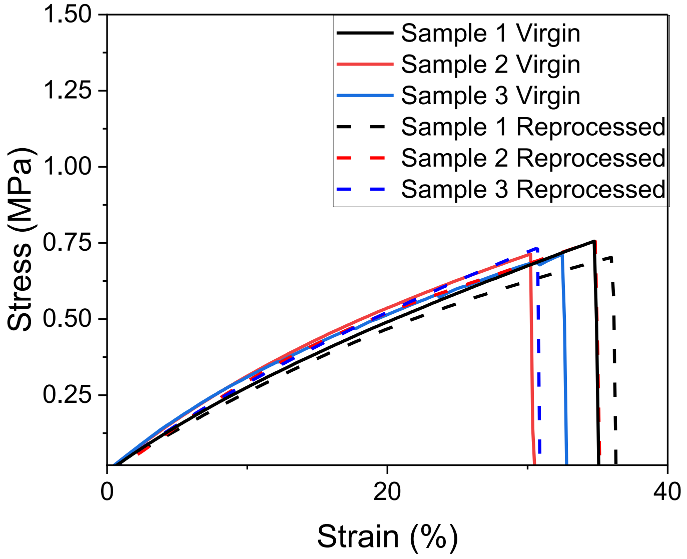

Figure S56. Stress-Strain plot for **Si-2(50-6)** virgin vs 3x reprocessed samples. Each sample was performed in triplicate.

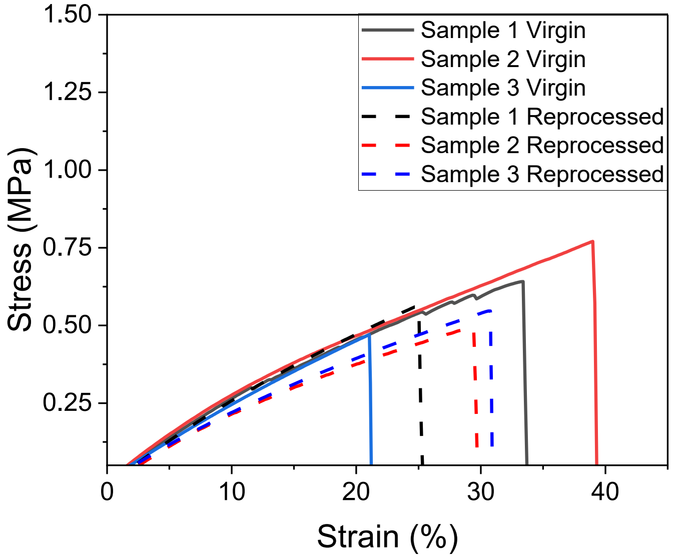

Figure S57. Stress-Strain plot for **Si-3(50-6)** virgin vs 3x reprocessed samples. Each sample was performed in triplicate.

Table S23. Si-2(50-6) thermomechanical data obtained from stress strain plots before and after reprocessing.

**Virgin**		**Young's Modulus (MPa)**	**Strain at Break (%)**	**Tensile Strength (MPa)**
	Sample 1	2.67	34.75	0.755
	Sample 2	3.1	30.2	0.713
	Sample 3	3.07	32.4	0.711
	**Average**	**2.947**	**32.45**	**0.726**
	**Error**	**0.14**	**1.31**	**0.014**

**Reprocessed 3x**		Young's Modulus (MPa)	Strain at Break (%)	Tensile Strength (MPa)
	Sample 1	2.63	35.97	0.703
	Sample 2	3.125	34.82	0.755
	Sample 3	2.928	30.5	0.731
	**Average**	**2.894**	**33.763**	**0.730**
	**Error**	**0.14**	**1.67**	**0.015**

Table S24. **Si-3(50-6)** thermomechanical data obtained from stress strain plots before and after reprocessing.

**Virgin**		**Young's Modulus (MPa)**	**Strain at Break (%)**	**Tensile Strength (MPa)**
	Sample 1	2.44	21.07	0.47
	Sample 2	2.66	33.4	0.64
	Sample 3	2.54	38.98	0.77
	**Average**	**2.547**	**31.15**	**0.627**
	**Error**	**0.06**	**5.29**	**0.09**

**Reprocessed 3x**		**Young's Modulus (MPa)**	**Strain at Break (%)**	**Tensile Strength (MPa)**
	Sample 1	2.24	30.76	0.547
	Sample 2	2.27	29.43	0.496
	Sample 3	2.67	25.07	0.563
	**Average**	**2.393**	**28.42**	**0.535**
	**Error**	**0.14**	**1.72**	**0.02**

DSC for reprocessed samples

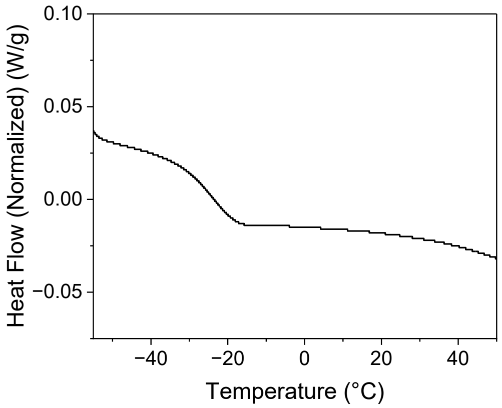

Figure S58. DSC thermogram for **Si-2(50-6)** reprocessed

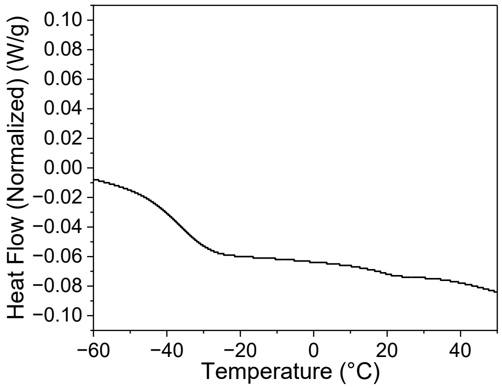

Figure S59. DSC thermogram for **Si-3(50-6)** reprocessed

Table S25. Glass transition temperature (Tg,onset; Tg,midpoint, and Tg,endpoint) for reprocessed polymer samples

Sample	Tg onset (˚C)	Tg midpoint (˚C)	Tg endpoint (˚C)
**Si-2(50-6) reprocessed**	-29.88	-23.94	-17.98
**Si-3(50-6) reprocessed**	-43.73	-36.02	-28.30

Stress Relaxation for Si-2(50-6) 3x reprocessed

It should be noted that for this sample, stress relaxation was performed using the DMA on the rectangular samples rather than the rheometer. A strain of 1% was applied onto the sample.

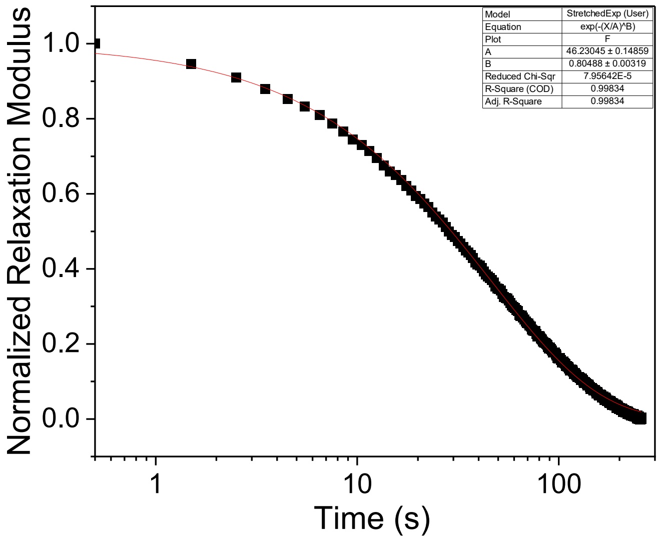

Figure S60. Normalized stress relaxation data for Si-2(50-6) reprocessed 3x at 70 ˚C.

Degradation Data

The ^{1}H NMR spectra of the 1M pTSA in THF solution was taken within the first 15 minutes of adding the polymer samples to the solution and then after 24 hours once all polymer networks had degraded. The NMR for all samples shows the presence of a peak around 0.21 ppm which can be attributed to methyl hydrogens from the silanol hydrolysis product.

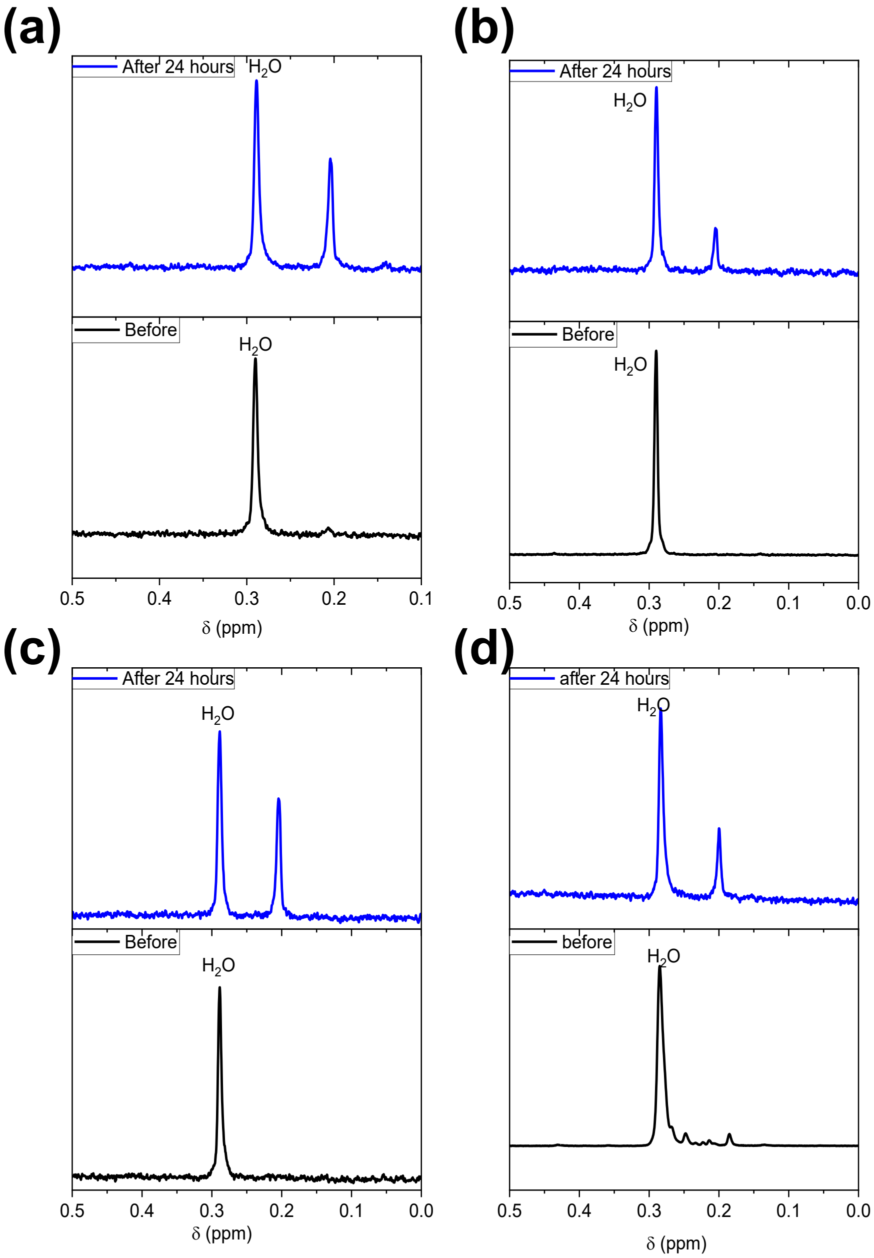

Figure S61. ^{1}H NMR of the THF solution before and after polymer degradation for **(a) Si-1(50-0)**, **(b) Si-2(50-0)**, **(c) Si-3(50-0)**, **(d) Si-4(50-0)**

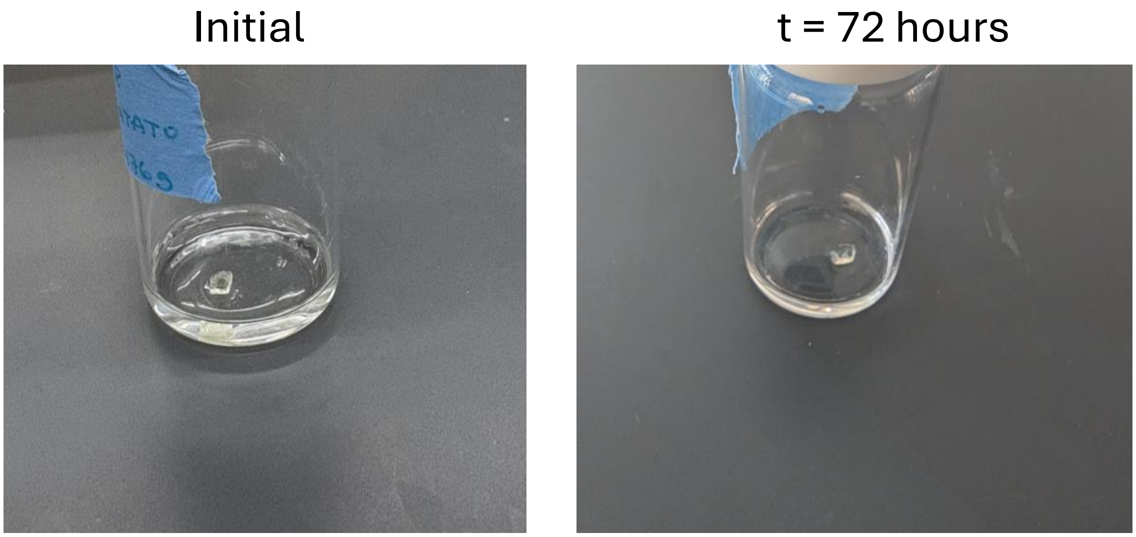

Figure S62. Pictures of a **Si-2(50-0)** in THF before and after 72 hours indicating no degradation of the polymer network in the absence of pTSA.
